# Classification of acute myeloid leukemia based on multi‐omics and prognosis prediction value

**DOI:** 10.1002/1878-0261.70000

**Published:** 2025-02-10

**Authors:** Yang Song, Zhe Wang, Guangji Zhang, Jiangxue Hou, Kaiqi Liu, Shuning Wei, Yan Li, Chunlin Zhou, Dong Lin, Min Wang, Hui Wei, Jianxiang Wang, Tao Cheng, Yingchang Mi

**Affiliations:** ^1^ State Key Laboratory of Experimental Hematology, National Clinical Research Center for Blood Diseases, Haihe Laboratory of Cell Ecosystem, Institute of Hematology & Blood Diseases Hospital Chinese Academy of Medical Sciences & Peking Union Medical College and Tianjin Institutes of Health Science China

**Keywords:** acute myeloid leukemia, classification, multi‐omics, prognosis

## Abstract

Acute myeloid leukemia (AML) is a heterogeneous cancer, making outcomes prediction challenging. Several predictive and prognostic models are used but have considerable inaccuracy at individual level. We tried to increase prediction accuracy using a multi‐omics strategy. We interrogated data from 1391 consecutive, newly diagnosed subjects with AML, integrating information on mutation topography, DNA methylation, and transcriptomics. We developed an unsupervised multi‐omics classification system (UAMOCS) with these data. UAMOCS provides a multidimensional understanding of AML heterogeneity and stratifies subjects into three cohorts: (a) UAMOCS1 [high lymphocyte activating 3 (*LAG3*) expression, chromosome instability, myelodysplasia‐related mutations]; (b) UAMOCS2 (monocytic‐like profile, immune suppression and activated angiogenesis and hypoxia pathways); and (c) UAMOCS3 [CCAAT enhancer binding protein alpha (*CEBPA*) mutations and MYC pathway activation]. UAMOCS distinguishes overall survival rates across the cohorts (TCGA *P* = 0.042; GSE71014 *P* = 0.043; ihCAMs‐AML, GSE102691 and GSE37642 all *P* < 0.001). The model's *C*‐statistic is comparable to the 2022 ELN risk classification (0.87 vs 0.82; *P* = 0.162), but offers a more nuanced distinction between intermediate‐ and high‐risk groups. When combined with high‐throughput drug sensitivity testing, UAMOCS can accurately predict sensitivity to azacitidine (AZA) and venetoclax. The UAMOCS system is available as an R package. The UAMOCS system has the potential to redefine AML subtypes, enhance prognostic predictions, and guide treatment strategies based on patients' immune status and expected responses to therapies.

## Graphical abstract

The Unsupervised AML Multi‐Omics Classification System (UAMOCS) integrates genomic, methylation, and transcriptomic data to categorize AML patients into three subtypes (UAMOCS1‐3). This classification reveals clinical relevance, highlighting immune and chromosomal characteristics, prognosis, and therapeutic vulnerabilities. *Ex vivo* screening of 17 drugs identified potential responses to AZA + Venetoclax treatment in AML patients.

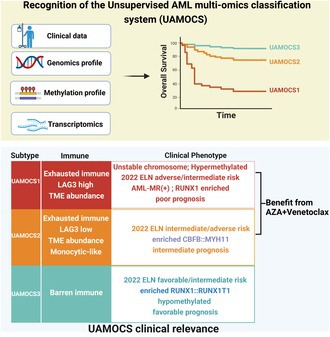

**Abbreviations**
AML: acute myeloid leukemia
AML‐MR: acute myeloid leukemia with myelodysplasia‐related changes
CBF‐AML: core binding factor‐acute myeloid leukemia
CEBPA: CCAAT/enhancer binding protein alpha
CNA: copy number alterations
CPI: Consensus Partitioning Index
EFS: event‐free survival
ELN: European LeukemiaNet
FAB: French–American–British Classification
HSCT: hematopoietic stem cell transplantation
IES: Immune Enrichment Score
lncRNA: long non‐coding RNA
ND: newly diagnosed
NTP: nearest template prediction
OS: overall survival
R/R: relapsed/refractory
SES: Stromal Enrichment Score
TF: transcription factor
TMB: tumor mutational burden
UAMOCS: Unsupervised AML Multi‐Omics Classification System

## 1 Introduction

The intrinsic heterogeneity of acute myeloid leukemia (AML) presents a multifaceted landscape, often resulting in diverse therapeutic outcome [1, 2]. This diversity of AML reflects the intricate interactions between genetic, molecular, and clinical determinants unique to each patient. The molecular profiles of gene mutations, chromosomal anomalies within AML considerably impacts disease progression, therapeutic response, and survival prognosis [2].

Traditional classification of AML is predominantly based on the genetic aberrations rather than morphological definitions [3]. Meanwhile, the use of transcriptomic gene expression profiling is increasingly becoming popular [4, 5, 6, 7]. Of note, Cheng et al. [8] identified transcriptome‐based subtypes and differentiation hierarchies in AML, showing their potential to improve classification and prognosis. Nonetheless, research at this singular omic scale has not fully deciphered the complex relationship between AML's molecular features and its clinical manifestations. Consequently, this complexity underscores the necessity for multi‐omics analyses to incorporate the synergistic interactions and communications among various molecular biomarkers.

In fact, certain studies have explored multi‐omics of AML, enhancing our understanding of the molecular attributes. For example, Bolouri et al. [9] highlighted the role of copy number alterations (CNA), mutations, miRNA, and DNA promoter hypermethylation as prognostic factors in pediatric AML. Jayavelu et al. [10] identified a specific AML cluster with high mitochondrial protein expression and confers poor outcome. Severens et al. [11] mapped AML gene expression and *ex vivo* drug responses. Recently, Wang et al. [12] conducted a multi‐omics study in AML, identifying four molecularly distinct subtypes with prognostic relevance. However, the study did not address whether this new classification provided additional insights beyond the current classification system. In addition, we integrated data from real‐world cohorts to thoroughly investigate multi‐omics correlations with key biological characteristics, including immune alterations, pathway involvement, clinical outcomes, and *ex vivo* drug responses.

In the present work, by employing a multitude of clustering algorithms, we have formulated an unsupervised AML multi‐omics classification system (UAMOCS), which delineates AML heterogeneity from multiple dimensions with clinical implications. UAMOCS not only identifies the immune status of different AML patients but also predicts their prognostication. More importantly, it identifies patients sensitive to Azacitidine (AZA) and Venetoclax, thereby guiding AML treatment regimens.

## 2 Materials and methods

### 2.1 Retrieval and preprocessing of TCGA AML profiling for initial consensus resemble

We acquired multi‐omic AML profiles from The Cancer Genome Atlas (TCGA) LAML cohort as discovery dataset. To avoid bias due to the highly favorable therapeutic response of acute promyelocytic leukemia (APL) to all‐trans retinoic acid (ATRA) and arsenic trioxide, APL samples were excluded from our AML classification analysis. This dataset includes 90 non‐APL cases, each with complete clinical information (survival time) and comprehensive multi‐omic data, including bulk RNA sequencing, genomic methylation profiles, and CNA profiles. These were accessed via the R package ‘TCGAbiolinks’ [13]. In our transcriptomic analysis, we categorized mRNAs, identified through the Vega database (https://vega.archive.ensembl.org/), and lncRNAs, annotated using the GENCODE 27 file, following the exclusion of genes with low expression. We adopted TPM (transcripts per million) values, transformed with log_2_ calculations, as the standard metric for gene expression. For somatic mutation data, we sourced comprehensive datasets from Firehose (http://www.firehose.org/).

DNA methylation data were processed using the ‘ChAMP’ R package [14] with comprehensive quality control steps. Probes with a detection *P*‐value > 0.01, fewer than three beads in at least 5% of the samples, non‐CpG probes, SNP‐related probes, multi‐hit probes, and probes located on sex chromosomes (X and Y) were excluded to ensure data reliability and minimize biases. To identify background methylation, normal peripheral blood data from the GSE51388 [15] dataset were used as a reference. As detailed in a previous study [16], which outlines specific criteria for demethylation, we evaluated the AML‐specific demethylation with two steps: firstly, we removed any background influences by excluding those sites in the AML samples that had a beta value > 0.8 in the normal samples. Secondly, we focused on sites with high variability (standard deviation > 0.2). A detailed list of 1597 demethylator genes used for clustering is now provided in Table S1. Somatic CNAs in TCGA's AML samples were analyzed using the GISTIC2.0 [17] algorithm with the thresholds of copy number amplifications/deletions being equal to ±0.3 (*q*‐value < 0.05).

### 2.2 Retrieval and preprocessing public AML data for validation

To validate our findings from a transcriptomic perspective, we sourced additional AML transcriptomic data (excluding APL cases) from the Gene Expression Omnibus (GEO) repository. We specifically selected GEO datasets that included detailed clinical data and RNA‐seq expression matrices. The datasets incorporated into our analysis were GSE37642 (*n* = 438), GSE71014 (*n* = 104), GSE14468 (*n* = 411), and GSE106291 (*n* = 250). To address potential batch effects across these cohorts, we employed the ‘sva’ package (version 3.29.1) in R, using the ‘ComBat’ algorithm [18].

### 2.3 Participants of ihCAMs‐AML cohort for validation

We conducted a retrospective analysis of a real‐world cohort of 98 *de novo* AML patients (non‐APL), including 80 newly diagnosed (ND) cases and 18 relapsed/refractory (R/R) cases, which served as the validation dataset (Table 1). This cohort, referred to as ‘ihCAMs‐AML’, comprised patients aged 14–70 years, with a median follow‐up period of 32 months (range: 1–65 months). Patients in the ihCAMs‐AML cohort were enrolled at the Institute of Hematology and Blood Diseases Hospital between March 2019 and September 2021. All participants provided written informed consent. The study was approved by the Ethics Committee of the Institute of Hematology & Blood Diseases Hospital (Ethical batch number: KT2020004‐EC‐2, NSFC 2022038‐EC‐2) and was conducted in accordance with the Declaration of Helsinki.

**Table 1:** Clinical characteristics in ihCAMs‐AML cohort (*N* = 98) under UAMOCS. ELN, European LeukemiaNet; HSCT, hematopoietic stem cell transplantation; ND, newly diagnosed; R/R, relapsed/refractory.

	Level	Overall	UAMOCS1	UAMOCS2	UAMOCS3	Overall *P*	UAMOCS1 vs. UAMOCS2 *P*‐value	UAMOCS1 vs. UAMOCS3 *P*‐value	UAMOCS2 vs. UAMOCS3 *P*‐value
*N*		98	23	38	37				
Disease status (%)	ND	80 (81.6)	18 (78.3)	30 (78.9)	32 (86.5)	0.625	1.000	0.635	0.577
	R/R	18 (18.4)	5 (21.7)	8 (21.1)	5 (13.5)				
Age (%)	≤ 55	80 (81.6)	15 (65.2)	32 (84.2)	33 (89.2)	0.058	0.163	0.054	0.768
	> 55	18 (18.4)	8 (34.8)	6 (15.8)	4 (10.8)				
Sex (%)	Female	47 (48.0)	9 (39.1)	18 (47.4)	20 (54.1)	0.529	0.717	0.390	0.728
	Male	51 (52.0)	14 (60.9)	20 (52.6)	17 (45.9)				
HSCT (%)	No	74 (75.5)	19 (82.6)	28 (73.7)	27 (73.0)	0.662	0.625	0.586	1.000
	Yes	24 (24.5)	4 (17.4)	10 (26.3)	10 (27.0)				
Status (%)	Alive	62 (63.3)	8 (34.8)	23 (60.5)	31 (83.8)	0.001	0.092	< 0.001	0.047
	Dead	36 (36.7)	15 (65.2)	15 (39.5)	6 (16.2)				
FAB classification (%)	M0	1 (1.0)	1 (4.3)	0 (0.0)	0 (0.0)	0.001	0.169	0.107	< 0.001
	M1	4 (4.1)	1 (4.3)	1 (2.6)	2 (5.4)				
	M2	35 (35.7)	8 (34.8)	5 (13.2)	22 (59.5)				
	M4	18 (18.4)	3 (13.0)	8 (21.1)	7 (18.9)				
	M5	40 (40.8)	10 (43.5)	24 (63.2)	6 (16.2)				
Cytogenetic group (%)	Adverse	18 (18.4)	8 (34.8)	8 (21.1)	2 (5.4)	0.044	0.244	0.007	0.136
	Favorable	21 (21.4)	2 (8.7)	9 (23.7)	10 (27.0)				
	Normal	59 (60.2)	13 (56.5)	21 (55.3)	25 (67.6)				
Cytogenetic abnormality (%)	Complex	7 (7.1)	4 (17.4)	1 (2.6)	2 (5.4)	0.002	0.162	0.031	0.002
	del(7)	1 (1.0)	1 (4.3)	0 (0.0)	0 (0.0)				
	inv(16)	9 (9.2)	1 (4.3)	7 (18.4)	1 (2.7)				
	MLL rearrangement	10 (10.2)	3 (13.0)	7 (18.4)	0 (0.0)				
	None	59 (60.2)	13 (56.5)	21 (55.3)	25 (67.6)				
	t(8;21)	12 (12.2)	1 (4.3)	2 (5.3)	9 (24.3)				
2022 ELN (%)	Adverse	33 (33.7)	15 (65.2)	14 (36.8)	4 (10.8)	0.001	0.097	< 0.001	0.023
	Favorable	46 (46.9)	5 (21.7)	16 (42.1)	25 (67.6)				
	Intermediate	19 (19.4)	3 (13.0)	8 (21.1)	8 (21.6)				

### 2.4 RNA‐seq and 850K methylation detection on ihCAMs‐AML cohort

A subset of patients within this cohort underwent DNA methylation analysis (*n* = 30, 16 R/R and 14 ND cases) and RNA sequencing (RNA‐seq, *n* = 98, 80 ND and 18 R/R cases). Total RNA was extracted from frozen bone marrow mononuclear cells of all participants using the RNA Assay Kit, a part of the Qubit^®^ 2.0 Fluorometer system (Life Technologies, Carlsbad, CA, USA). RNA samples with RIN values above 7.0 were used for library preparation. Libraries were generated using the NEBNext^®^ Ultra™ RNA Library Prep Kit for Illumina^®^ (New England Biolabs, Ipswich, MA, USA) following the manufacturer's protocol. mRNA was enriched, fragmented, and converted into cDNA. The resulting cDNA libraries were end‐repaired, adenylated, and ligated with adapters. PCR amplification was performed to generate final cDNA libraries. Libraries were sequenced on an Illumina NovaSeq 6000 platform (Illumina, San Diego, CA, USA) using paired‐end reads of 150 bp. Paired‐end reads were mapped to the GRCh37 human genome reference by hisat2 v2.1.0 [19]. Gene annotation files were downloaded from Ensembl (http://www.ensembl.org/) for subsequent evaluation of gene expression levels.

Methylation analysis of genes was executed using the comprehensive 850K methylation assay. Genomic DNA was meticulously extracted from bone marrow samples utilizing the DNeasy Blood and Tissue Kit, following the Qiagen, Hilden, North Rhine‐Westphalia, Germany protocol to ensure high‐quality DNA isolation. For each subject, an aliquot of 1 μg of DNA was subjected to bisulfite conversion using the Zymo Research EZ Methylation Kit, Zymo Research Corporation, Irvine, CA, USA a critical step for accurate methylation analysis. The microarray experiment was carried out in accordance with the detailed specifications provided by the manufacturer of the Illumina Methylation 850K kit (Illumina, San Diego, CA, USA), ensuring a standardized and reliable assessment of DNA methylation.

### 2.5 Targeted exome sequencing and mutation calling on ihCAMs‐AML cohort

Targeted exome sequencing was carried out on 96 eligible patients in ihCAMs‐AML (*n* = 96, R/R 16 and ND 80 cases), using a customized panel targeting 267 hotspot mutations associated with malignant hematologic disorders (Table S2). The sequencing process was conducted using the Illumina NovaSeq 6000 platform. To ensure the quality of the sequencing data, the fastp tool (version 0.23.2, https://github.com/OpenGene/fastp) [20] was utilized for removal of adapter sequences and low‐quality reads from the raw fastq data. The alignment of the sequencing data and the calling of mutations were executed using the DRAGEN pipeline (version 3.10.4). Candidate somatic mutations are defined as variants with a variant allele frequency (VAF) > 0.5%. The final candidate variants were manually verified using the Integrative Genomics Viewer (IGV) [21]. Additionally, the study employed the pindel tool (version v0.2.5b8) [22] and the flt3_itd_ext tool (version 1.1) [23] specifically to detect FLT3 internal tandem duplication (ITD) alleles, focusing on exons 13–15.

### 2.6 *Ex vivo* drug screening analysis on ihCAMs‐AML cohort

In this study, we obtained 62 (18 R/R cases and 44 ND cases) primary bone marrow samples from the ihCAMs‐AML cohort, including data from 62 cases for the 100% PPC concentration inhibition assay, and from 22 ND cases for drug sensitivity testing across seven concentration gradients. The samples were preserved in EDTA anticoagulant tubes and transported under 4 °C conditions. And the samples were stored in cold chain transportation for no more than 72 h. We obtained primary cancer cells by density gradient centrifugation. And then we utilized a specialized complete culture medium for AML (Human Acute Myeloid Leukemia Cell Medium, Precedo, PRS‐AMLM) to amplify cancer cells *in vitro*. AML cells were seeded and cultured in 384‐well opaque culture plates (Nest, 761601) at a density of 5000 cells/well, with triplicate wells for each drug. The cells were incubated in a 5% CO_2_ incubator at 37 °C for 72 h. Drug administration (0.1 μL per well) was performed using a JANUS automated workstation (Perkin Elmer Inc., Wellesley, MA, USA), and 10 μL of CellCounting‐Lite 2.0 luminescent cell viability assay reagent was added to each well. After 10 min, the fluorescence, represented by relative luminescence units (RLU), was measured using an EnVision plate reader (Perkin Elmer Inc., Wellesley, MA, USA). The inhibition rate was calculated as follows: inhibition rate = 100% − (RLUDrug − RLUBackground)/(RLUDMSO − RLUBackground) × 100%. The specifics regarding the experimental drugs and their concentrations are detailed in the Table S3.

### 2.7 Multi‐omics consensus ensemble

In order to conduct a multi‐omics classification of non‐APL cases from the TCGA cohort, we calculated the optimal assembly numbers using the cumulative probability index (CPI) and gap statistics, alongside the classification of AML subtypes. Following this, we used 10 advanced multi‐omics methods, including iClusterBayes, moCluster, CIMLR, IntNMF, ConsensusClustering, COCA, NEMO, PINSPlus, SNF, and LRA, as part of a consensus ensemble approach [24]. These methods provide complementary insights into the data, and their results were integrated using the MOVICS R package, leading to a more robust and reliable final classification model [25]. To enhance the selection of candidates for the formation of the UAMOCS, we established specific thresholds for various molecular data types. For continuous data, we identified the top 1000 features exhibiting high standard deviation, which encompassed mRNAs, long non‐coding RNAs (lncRNAs), and demethylated DNA methylation sites. In the case of binary data, specifically somatic mutations, we selected those mutations present in at least 3% of the samples, resulting in a total of 65 identified mutations (Table S4).

### 2.8 Tumor microenvironment profiling estimation

To assess the TME composition of each AML case, we used the R package MCPcounter [26], which provides abundance scores for eight immune populations (T cells, CD8+ T cells, cytotoxic lymphocytes, natural killer cells, B cell lineage, monocytic lineage, myeloid dendritic cells, and neutrophils) and two stromal populations (endothelial cells and fibroblasts). To determine the functional orientation of TME, we used signatures derived from the literature [27] including immunosuppression (CXCL12, TGFB1, TGFB3, and LGALS1), T‐cell activation (CXCL9, CXCL10, CXCL16, IFNG, and IL15), T‐cell survival (CD70 and CD27), Tregs (FOXP3 and TNFRSF18), major histocompatibility complex class I (HLA‐A, HLA‐B, HLA‐C, HLA‐E, HLA‐F, HLA‐G, and B2M), myeloid cell chemotaxis (CCL2), and tertiary lymphoid structures (TLSs) (CXCL13). We also collected four immune suppression‐associated signatures including tumor‐infiltrating Tregs, myeloid‐derived suppressor cell (MDSC), cancer‐associated extracellular matrix (C‐ECM), and Wnt/TGF‐β. Then, we quantified the expression levels of six Immune Checkpoint Inhibitors (ICIs), including PDCD1, CD274, PDCD1LG2, HAVCR2, CTLA4, and LAG3 [28]. The estimation of stromal cell content within the AML BMMCs was defined by the Stromal Enrichment Score (SES) and Immune Enrichment Score (IES), assessed using the R package ‘ESTIMATE’ (version 1.0.13) [29]. Scores for each signature were calculated as the geometric mean of signature expression.

### 2.9 Differential analysis and functional enrichment

To deepen our understanding of the biological distinctions among UAMOCS phenotypes, we analyzed differentially methylated probes (DMPs) using the ‘ChAMP 2.18.2’ R package [14], employing its default settings. Additionally, we identified differentially expressed genes (DEGs) across the three subtypes using the ‘limma’ package [30]. Genes exhibiting a false discovery rate (FDR) < 0.05 and a log_2_ fold change (FC) of 1 or greater were identified as differentially expressed between the groups. For GSEA (Available at http://www.broadinstitute.org/gsea/index.jsp) based on transcriptome expression data, we prepared a preranked gene list according to the descending ordered log_2_FoldChange value derived from differential expression analysis; we then leveraged the R package clusterProfiler [31] to determine functional enrichment based on the Hallmark pathway.

### 2.10 Prediction of UAMOCS phenotypes

To further validate the UAMOCS phenotypes, we employed the Nearest Template Prediction (NTP) method [32], on *Z*‐score normalized gene expression data across five independent cohorts, including four GEO datasets and the ihCAMs‐AML cohort.

### 2.11 Regulon analysis

We used R package ‘RTN 2.20’ [33] to reconstruct transcriptional regulatory networks (regulons) including specific transcription factors (TFs) relevant to AML [34] as well as chromatin remodeling markers identified in solid tumor [35]. Specifically, mutual information analysis and Spearman rank‐order correlation deduced the possible associations between a regulator and all potential target from the transcriptome expression profile, and permutation analysis was utilized to erase associations with an FDR > 0.05. Bootstrapping strategy removed unstable associations through one thousand times of resampling with consensus bootstrap > 95%. Data processing inequality filtering eliminated the weakest associations in triangles of two regulators and common targets. Individual regulon activity was estimated by two‐sided GSEA, as previously described by Lu et al. [24].

### 2.12 Small gene panel training

In order to identify the most representative gene panel in our 150‐gene signature to accelerate translational medicine, we incorporated data from 1391 cases across six independent datasets. Our approach primarily integrated the Partitioning Around Medoids (PAM) score into the evaluation of subtype dependency genes. For each gene, the PAM score indicates its positive or negative correlation with gene expression in each subtype. From these identified signatures, we selectively focused on genes that showed a positive association with each class. To maintain a balanced signature size, we set a threshold of 0.2 for UAMOCS1 and UAMOCS3, and 0.5 for UAMOCS2, ending up with 5 genes for UAMOCS1, 6 genes for UAMOCS2, and 5 genes for UAMOCS3. To test the predictive performance of the refined 16‐gene panel, we employed the random forest algorithm using the ‘randomForestSRC’ R package (v3.2.1, http://cran.r‐project.org/web/packages/randomForestSRC/index.html). To reduce over‐fitting and enhance robustness of the random forest classifier, we built 10 000 classifiers with randomly extracted 50% of the samples from each cohort. These classifiers were further applied in the all samples in six cohorts independently to calculate an accuracy distribution of the 16‐gene panel.

### 2.13 Statistical analyses

All statistical analyses were implemented using R (version 4.21, R Foundation for Statistical Computing, Vienna, Austria). Continuous parameters among the UAMOCS molecular subtypes were compared using the Kruskal–Wallis test, while categorical variables were analyzed with Fisher's exact test. Kaplan–Meier curves were employed for survival analysis among the three subtypes. For unadjusted comparisons, a two‐sided *P* < 0.05 was considered statistically significant.

## 3 Results

### 3.1 Multi‐omics ensemble identifies three UAMOCS phenotypes

**Fig. 1:**
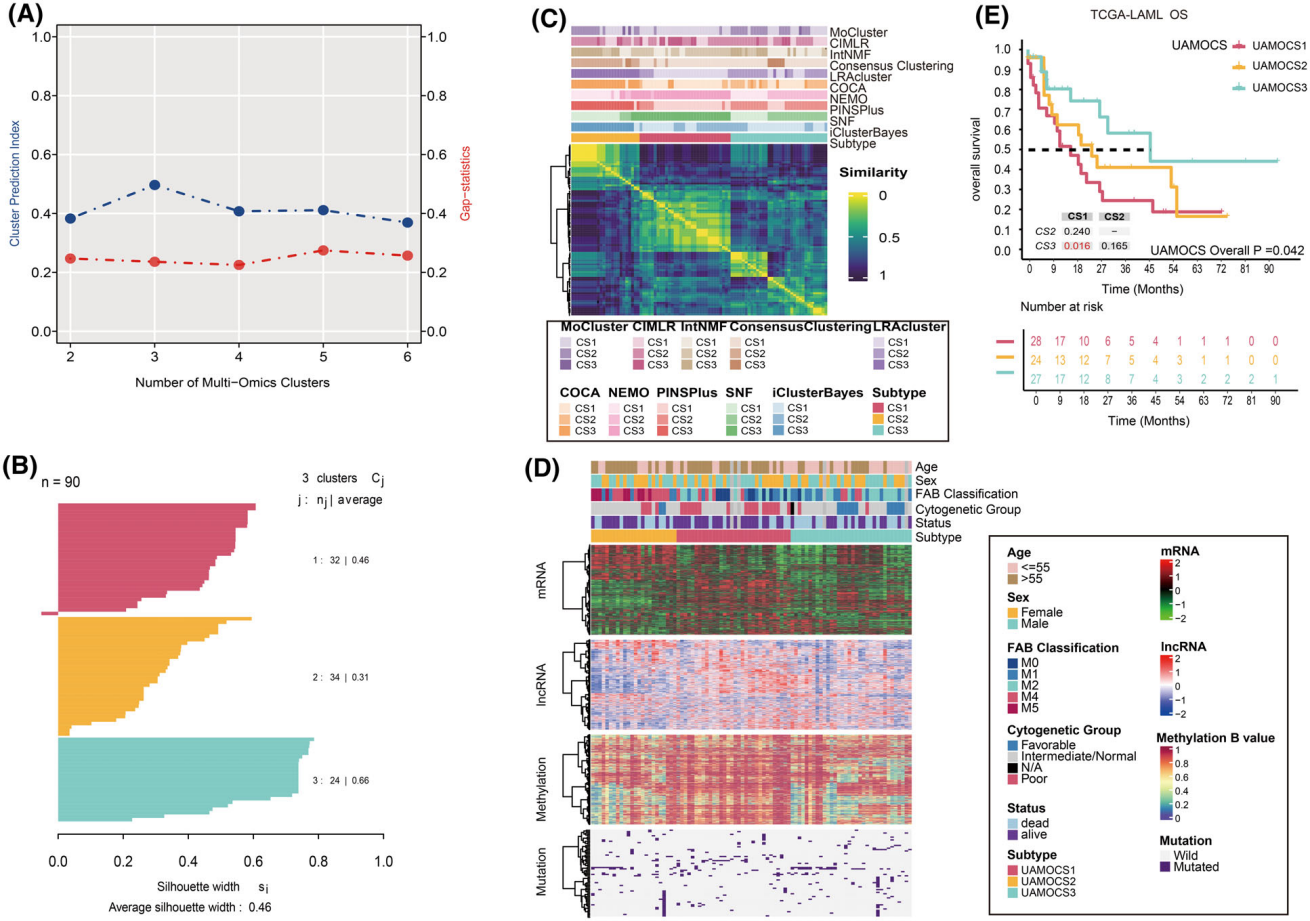
Recognition and clinical relevance of Unsupervised AML Multi‐Omics Classification System (UAMOCS) in The Cancer Genome Atlas (TCGA) database. (A) Consensus Clustering Statistics in TCGA‐LAML Cohort, involving two key statistical indices used for consensus clustering in the TCGA‐LAML cohort, computed using the R package ‘MOVICS’. The Consensus Partitioning Index (CPI) is depicted by the blue line, where a higher CPI value signifies enhanced stability and reliability of the identified clusters. The red line represents the gap statistic, which determines the optimal number of clusters by contrasting the change in within‐cluster dispersion with an expected null reference data distribution. A peak in the gap statistic indicates the most appropriate number of clusters. (B) Silhouette Plot for Consensus Clustering shows the consensus clustering effect of the three subtypes, with an average silhouette score of 0.46. (C) Consensus Matrix Similarity Heatmap Utilizing 10 Clustering Algorithms: This heatmap represents the consensus matrix similarity, derived from the application of 10 commonly used clustering algorithms. (D) The consensus heatmap displays the transcriptomic profiling, including mRNA and Long Non‐Coding RNA (lncRNA) expression, methylation beta values, and mutations, organized from top to bottom. (E) The Kaplan–Meier overall survival (OS) curves compare across the three molecular subtypes in TCGA cohort. Overall *P*, Fisher exact test; pairwise *P*, Log‐rank test.

Multi‐omics profiles of 90 AML patients from the TCGA‐LAML cohort were analyzed. Taking into account two clustering statistics and corresponding silhouette scores, we determined the three optimal clusters among 90 AML patients from the TCGA database (Fig. 1A,B). A consensus ensemble generated from 10 multi‐omics integrative clustering approaches identified three robust UAMOCS subtype (Fig. 1C), which presented with distinctive molecular patterns across transcriptome expression, epigenetic methylation, and somatic mutation (Fig. 1D). Specifically, 35.5% (32/90) of AML patients were classified as UAMOCS1, 26.6% (24/90) as UAMOCS2, and 37.8% (34/90) as UAMOCS3. We compared the prognosis of the three UAMOCS subtypes, UAMOCS3 showing better OS, UAMOCS1 with worse prognosis, and the median OS time gradually decreasing from UAMOCS3，UAMOCS2 to UAMOCS1 (45 vs. 27 vs. 18 months, *P* = 0.042, Fig. 1E).

**Fig. 2:**
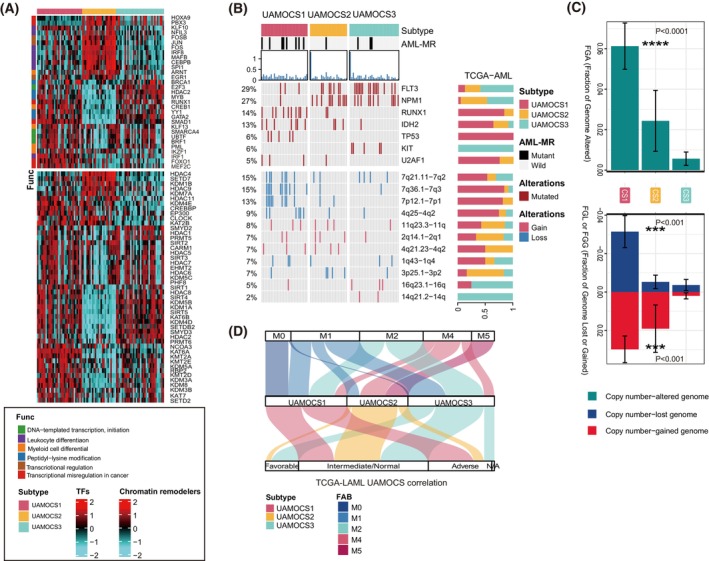
Delineation of UAMOCS classification system in The Cancer Genome Atlas (TCGA) database. (A) The regulon activity heatmap highlights the distinct patterns of TCGA‐LAML regulon activity among the three UAMOCS subtypes. It includes 31 transcription factors (TFs) in the top panel and potential regulators associated with chromatin remodeling in the bottom panel. (B) The waterfall plot displays key genetic characteristics across the three acute myeloid leukemia (AML) UAMOCS phenotypes. It includes the prevalence of acute myeloid leukemia with myelodysplasia‐related changes (AML‐MR) mutations, tumor mutational burden (TMB) expressed in mutations/Mb, frequency of commonly mutated genes, and the alteration ratio of the genome. (C) The bar plots show the fraction of the genome altered (FGA) and the fraction of genome lost or gained (FGL/FGG) among three TCGA UAMOCS subtypes. The top plot compares FGA among three UAMOCS subtypes, while the bottom plot compares FGL (blue), and FGG (red). The error bars indicate standard error mean (SEM). Statistical significance was determined using Kruskal–Wallis test, with *** indicating *P* < 0.001 and **** indicating *P* < 0.0001. *P* values compare the differences in FGA, FGL, and FGG across the three UAMOCS subtypes, respectively. (D) The Sankey plot here demonstrates a strong correlation between UAMOCS categorization, cytogenetic abnormalities, and the French–American–British (FAB) classification system in TCGA‐LAML cohort.

To delve deeper into the transcriptomic variations, we conducted an analysis of regulons for AML‐specific TFs and potential regulators that are pertinent to cancerous chromatin remodeling (Fig. 2A). Notably, TFs like *IKZF1*, *IRF1*, *FOXO1*, and *MEF2C*, which are implicated in the progression to a preleukemic state, were distinctly prevalent in UAMOCS1. Regulon activity profiles that were associated with cancerous chromatin remodeling highlighted other possible differential regulatory patterns among the UAMOCS system, indicating that epigenetically driven transcriptional networks might be important differentiators of these molecular subtypes.

The potential epigenetic differences among the subtypes might be further supported by the different methylation landscape. Specifically, we focused on CpG sites that lose methylation (i.e., ‘demethylate’) in AML. These probes were previously reported to have implications in AML pathogenesis [16]. We selected probes with high methylation levels in normal peripheral blood but variable methylation across the AML cohort. We found that UAMOCS2 group showing demethylator phenotype, consistent with its relatively poor prognosis while UAMOCS1 had generally hypermethylation pattern (Fig. 1D).

In the TCGA‐LAML dataset, AML patients generally presented with a low tumor mutational burden (TMB), with no significant variations noted among the three subtypes (Fig. 2B and Fig. S1A). Meanwhile, we found a frequent occurrence of AML‐MR mutations in UAMOCS1 compared to other subtypes (32.3%, vs. 3.7% vs. 9.4%, *P* < 0.001). Additionally, among genes mutated in more than 5% of cases, UAMOCS1 exhibited significantly fewer *FLT3‐ITD* (9.4%, vs. 27.2% vs. 47.1%, *P* = 0.003) and *NPM1* mutations (3.1%, vs. 45.8% vs. 32.4%, *P* < 0.001), while higher mutations of *RUNX1* (31.2%, vs. 8.3% vs. 0, *P* < 0.001), *IDH2* (25%, vs. 12.5% vs. 2.9%, *P* < 0.001), and *TP53* (15.6%, vs. 0 vs. 0, *P* = 0.005).

We employed the GISTIC 2.0 algorithm to analyze the genomic copy number distribution (Fig. S1B). Notably, UAMOCS1 had markedly the fraction of the genome altered (FGA) than other subtypes (Fig. 2C), as evidenced by significantly higher incidences of copy number loss or gain (Fig. S1C,D, both, *P* < 0.001). Specifically, deletions in regions chromosome 7q (7q21, 7q36, and 7p12) were predominantly observed in the UAMOCS1 group (Fig. 2B). UAMOCS1 was also characterized by poor and intermediate cytogenetic stratifications and while UAMOCS2 typically displayed an intermediate or normal cytogenetic risk profile (Fig. 2D). Nevertheless, the UAMOCS system may extend the capability of cytogenetic risk stratification as UAMOCS subclassification also correlated with FAB classification, monocytic cells (FAB‐M4/M5) were chiefly categorized within the UAMOCS2 subgroup, correlating with an intermediate prognosis.

### 3.2 Reproducibility of the UAMOCS system using transcriptomic signatures

**Fig. 3:**
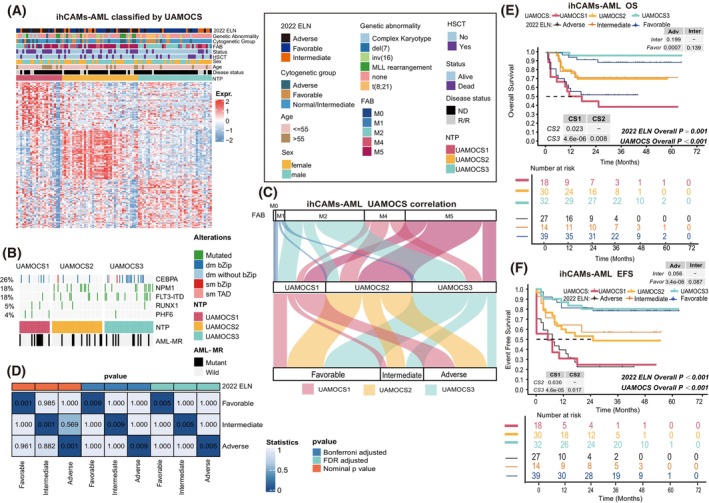
Molecular features of UAMOCS in ihCAMs‐AML cohort. (A) The heatmap illustrates the transcriptomic profiles and basic clinical characteristics associated with UAMOCS subtyping, determined via the nearest template prediction (NTP) method in the ihCAMs‐AML cohort. ELN, European LeukemiaNet; HSCT, hematopoietic stem cell transplantation; ND, newly diagnosed; R/R, relapsed/refractory. (B) The waterfall plot displays the significant somatic mutations across the three AML UAMOCS phenotypes in the ihCAMs‐AML cohort. dm bZIP: Biallelic mutations of *CEBPA* C‐terminal DNA‐binding or basic leucine zipper region; dm without bZIP: Biallelic mutations of *CEBPA* excluded C‐terminal DNA‐binding or basic leucine zipper region; sm bZIP; monoallelic mutations of *CEBPA* C‐terminal DNA‐binding or basic leucine zipper region; sm TAD: monoallelic mutations of *CEBPA* N‐terminal transactivation domains. (C) The Sankey plot here demonstrates a strong correlation between UAMOCS categorization, cytogenetic abnormalities, and the 2022 ELN system in ihCAMs‐AML cohort. (D) Correlation Submap illustrates the transcriptional similarity between the TCGA and ihCAMs‐AML cohorts based on different cytogenetic abnormality classification. (E) The Kaplan–Meier survival curve compares the overall survival differences among the ihCAMs‐AML cohort under the UAMOCS and 2022 ELN classifications. The horizontal dashed line represents the median survival, corresponding to the point where 50% of patients remain alive. (F) The Kaplan–Meier survival curve compares the Event‐free survival (EFS) differences among the ihCAMs‐AML cohort under the UAMOCS and 2022 ELN classifications. Adv, adverse; Favor, favorable; Inter, intermediate; Overall *P*, Fisher exact test; pairwise *P*, log‐rank test.

Given the prevalent use of transcriptome‐level data in cancer research, we identified 50 unique mRNAs significantly upregulated in distinct UAMOCS subtypes from the LAML‐TCGA cohort separately (Table S5). This led to the development of a comprehensive 150‐gene signature, intricately designed to predict UAMOCS subtypes from four published external datasets (Fig. S2A–D) and an additional real‐world cohort comprising of 98 cases, named ‘ihCAMs‐AML’ (Fig. 3A). Although multi‐omics profiling was initially planned for this cohort, substantial data loss due to sample quality issues posed technical challenges for multi‐omics integration. Consequently, each sample from these five datasets was classified into one of the UAMOCS classification system subtypes based on the 150 transcriptomic signatures, using the NTP algorithm.

In the ihCAMs‐AML cohort, we noted that the expression profiles of potential TFs involved in oncogenic chromatin remodeling were consistent with those observed in the TCGA cohort (Fig. S3A), further demonstrating the role of epigenetics in shaping the different subtypes within our UAMOCS system. In addition, we performed unsupervised demethylation analysis in 43 cases with profiled DNA methylation data. Consistently, the demethylated subtype enriched in predicted UAMOCS2 based on transcriptomic profiles, and the predicted UAMOCS1 showed generally hypermethylation pattern (Fig. S3B), suggesting the stable interaction between transcriptome and epigenome in shaping aggressiveness in AML.

Furthermore, the distribution of traditional cytogenetic stratification, genetic alterations, and FAB classification among UAMOCS were consistent with the TCGA‐LAML discovery dataset (Fig. 3A–C). Our investigation also uncovered that UAMOCS2 subgroup predominantly enriched monocytic features (FAB‐M4/M5) which were correlated with Abbas et al.'s findings [36]. Reinforcing this finding, we focused on 29 AML cell lines derived from the CCLE (Cancer Cell Line Encyclopedia) database that had available RNA‐seq data, and we classify these cell lines into one of our UAMOCS subtypes by NTP algorithm. Notably, monocytic AML cell lines, including THP1, MV411, and MOLM13, were predominantly classified into this subgroup (Fig. S3C).

In UAMOCS1 of ihCAMs‐AML cohort, we observed more adverse abnormalities, such as MLL rearrangement, del (7), and complex karyotype (Fig. 3A). All cases with AML‐MR mutations were categorized, and we further discovered that patients in UAMOCS1 tend to have more AML‐MR mutation genes (Fig. S3D). This analysis also revealed that mutations in splicing factors such as *U2AF1* and *RUNX1* were also predominantly found in this subtype. Consistently, we found *RUNX1* alterations were particularly enriched in UAMOCS1 in GSE37642 and GSE106291 (both, *P* < 0.001). The *CEBPA‐*bZIP mutation (reflecting its favorable prognosis) was notably more prevalent in the UAMOCS3 group within the ihCAMs‐AML cohort, which also aligns with the findings from the TCGA‐LAML dataset (Fig. 3B). The stratification of ihCAMs‐AML and TCGA‐LAML patients shows consistent patterns under UAMOCS, with similar proportions in genetic prognostic grouping, indicating that the system is stable (Fig. 3D).

The UAMOCS system's predictions in external cohorts highlighted its effectiveness in distinguishing survival outcome, as evidenced by significant OS results in ihCAMs‐AML (*P* < 0.001), GSE102691 (*P* < 0.001), GSE71014 (*P* = 0.043), and GSE37642 (*P* < 0.001) (Fig. 3E and Fig. S4A–C).

### 3.3 Comparison of UAMOCS and ELN stratification: providing a more reasonable understanding of core binding factor (CBF) AML

2022 ELN is a gold standard risk classification for AML patients. We compared the predictive value of UAMOCS for AML prognosis with 2022 ELN risk classification. The predictive power of the two criteria is comparable: OS *C*‐index (UAMOCS 0.87, ELN 0.82, *P* = 0.162) and Event‐free survival (EFS) *C*‐index (UAMOCS 0.8, ELN 0.82, *P* = 0.64).

Of note, our findings suggest that UAMOCS provides a more refined distinction between intermediate‐risk and adverse groups in the ihCAMs‐AML cohort compared to ELN 2022, indicating a trend in this direction (Fig. 3E,F). This was evident in both OS (UAMOCS1 vs. UAMOCS2 *P* = 0.023, ELN Adverse vs. Intermediate, *P* = 0.199), EFS (UAMOCS1 vs. UAMOCS2 *P* = 0.030, ELN Adverse vs. Intermediate, *P* = 0.056).

**Fig. 4:**
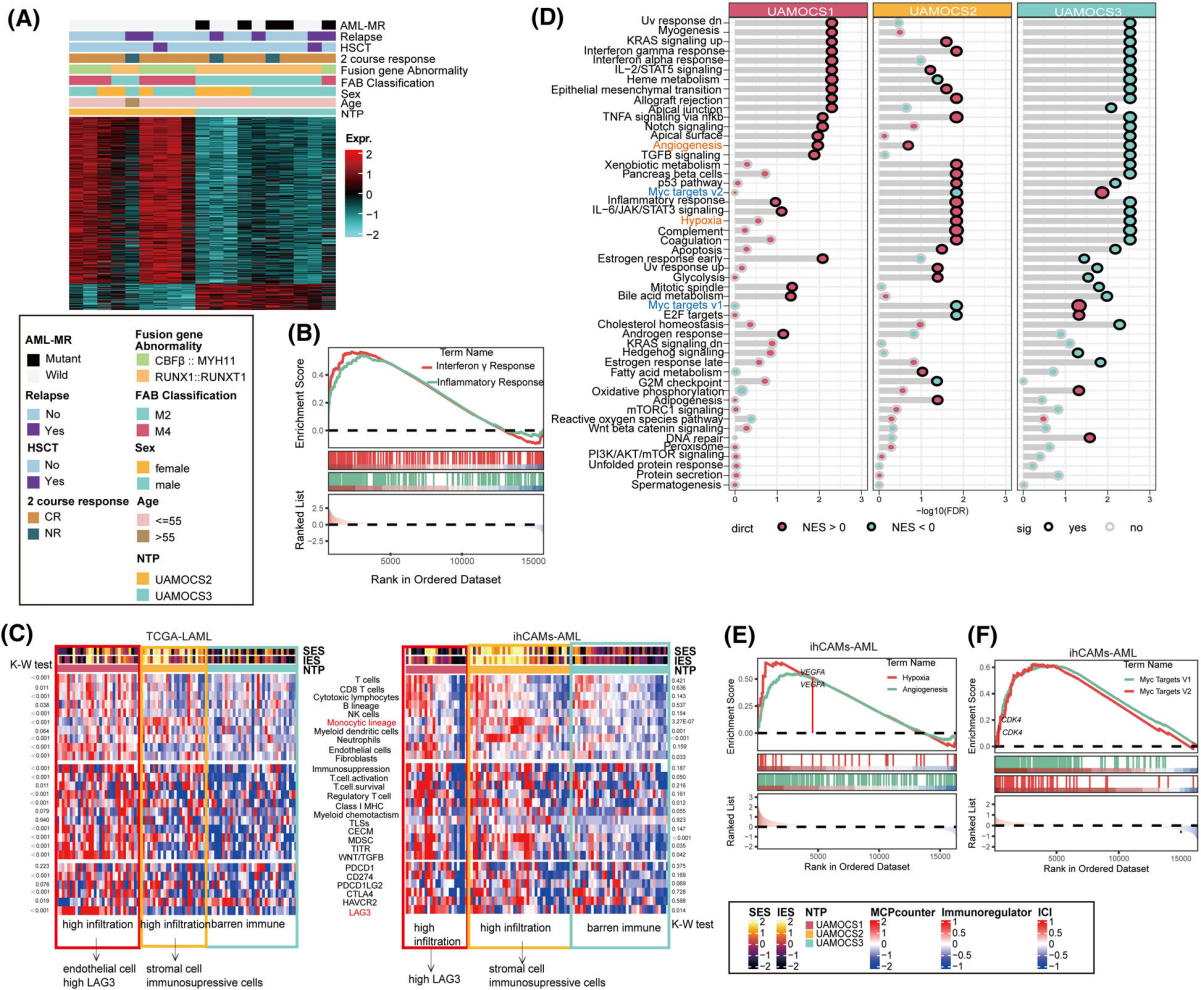
Characteristics of TCGA and ihCAMs‐AML classed by UAMOCS. (A) CBF‐AML Transcriptional Profiling and Clinical Comparison: This heatmap compares transcriptional profiling and clinical parameters between UAMOCS2 and UAMOCS3 in the context of CBF‐AML. CR, complete remission; NR, no remission. (B) Upregulated signaling pathways of UAMOCS2 vs. UAMOCS3 in CBF‐AML of ihCAMs‐AML cohort. (C) The heatmap showcases the immune landscape of UAMOCS in TCGA (left panel) and ihCAMs‐AML (right panel): It sequentially presents immune and stromal scores, proportions of various immune cells, key immune cell regulators, and markers associated with immune exhausting. Red text highlights UAMOCS1 features associated with the increased expression of exhaustion marker LAG3, and UAMOCS2, which is characterized by an aggregation of monocyte‐like cells. These red text features are consistently validated across multiple datasets. C‐ECM, cancer‐associated extracellular matrix; ICI, immune checkpoint inhibitors; IES, Immune Enrichment Score; MDSC, myeloid‐derived suppressor cells; SES, Stromal Enrichment Score; TITR, tumor‐infiltrating Tregs; TLS, tertiary lymphoid structures. (D) The GSEA dot plot for hallmark pathways illustrates the changes in biological pathways among the three UAMOCS subtypes within the TCGA cohort. UAMOCS2 (orange text) is enriched in angiogenesis and hypoxia‐related pathways, while UAMOCS3 (blue text) is enriched in MYC target pathways. These findings are consistently validated across multiple datasets. (E, F) The GSEA heatmap shows the distinct upregulated pathways unique to UAMOCS2 (E) and UAMOCS3 (F) within the ihCAMs‐AML cohort which was used to validate the findings from TCGA‐LAML.

In the ihCAMs‐AML cohort, the overall complete remission (CR) rate was 85.7% (84/98). When focusing only on ND patients, the induction therapy remission rates among the three subtypes were 27.8% (5/18), 60% (18/30), and 56.2% (18/32) (*P* = 0.074), indicating that the UAMOCS classification did not significantly impact treatment response (Table S6). Despite the lack of a clear trend in the overall *P*‐value, CR rates were lowest in UAMOCS1, intermediate in UAMOCS3, and highest in UAMOCS2. Notably, CBF‐AML cases were enriched in the UAMOCS2 and UAMOCS3 subtypes, aligning with their association with favorable prognosis. All CBF‐AML patients in the cohort belonged to these two subgroups, including *RUNX1::RUNX1T1* (*n* = 11) and *CBFβ::MYH11* (*n* = 8). Specifically, UAMOCS2 included 7 ND *CBFβ::MYH11* and 2 R/R *RUNX1::RUNX1T1* cases, while UAMOCS3 comprised 9 *RUNX1::RUNX1T1* cases (ND = 8, R/R = 1) and 1 R/R *CBFβ::MYH11* case (Fig. 4A and Table S7). These classifications were consistently observed in the GSE106291 dataset (Fig. S2B).

Further analysis of ND CBF‐AML cases in ihCAMs‐AML cohort revealed that UAMOCS2 included seven patients with *CBFβ::MYH11*, all of whom achieved CR after one induction chemotherapy cycle, whereas UAMOCS3 included eight patients with *RUNX1::RUNX1T1* fusions, none of whom achieved CR. This disparity in remission rates contributed to the higher CR rates observed in UAMOCS2 after one cycle of induction therapy. Furthermore, CBF‐AML patients in the UAMOCS2 group demonstrated a lower relapse rate compared to those in UAMOCS3 (22.2% vs. 60%, *P* = 0.04). However, due to the relatively small proportion of CBF cases in the ihCAMs‐AML cohort, this trend did not significantly impact the overall CR rate and did not influence the survival outcomes (OS and EFS) across the three UAMOCS subtypes.

Further analysis revealed that immune‐inflammatory pathways, such as the inflammatory response and interferon‐gamma response, were upregulated in the UAMOCS2 subgroup compared to UAMOCS3 (Fig. 4B). This observation aligns with immune subtyping within AML [37], indicating that CBF‐AML patients who respond favorably to treatment may share Interferon‐γ‐related pathways.

Also, CBF‐AML patients with MR mutations primarily clustered in the UAMOCS3 category, yet this did not significantly affect overall survival (*P* = 0.92), suggesting that MR mutations may not critically alter prognosis as discussed in CBF‐AML in 2022 ELN recommendations [2]. This finding highlights the prognostic relevance of the CBF classification in AML.

### 3.4 Different immune profiles across UAMOCS system

Recognizing the critical influence of immunity in the progression of AML, we speculated that bone marrow microenvironment in different UAMOCS subtypes might present unique features. Based on the analysis of suppressive immune cell and ICI marker expression, patients fell into two distinct categories in UAMOCS classification system. Type I, UAMOCS1, and UAMOCS2 were characterized by a high immune cell infiltration with exhaustion phenotype (Fig. 4C). UAMOCS1 was distinguished by T cells and endothelial cells enrichment, along with an increased expression of the exhaustion marker *LAG3*. UAMOCS2 exhibited an enrichment of stromal and immunosuppressive cells, including MDSCs and TITR (tumor‐infiltrating T regulatory cell), and *LAG3* low expression. Type II, UAMOCS3 was identified as the ‘Barren immune’ group and showed a pattern of low immune cell infiltration. The identified immune profiles could be reproduced in AML patients across other four eternal cohorts (Fig. S5A).

These comprehensive analyses across various datasets lend robust support to our findings from the real‐world cohort. The distinct phenotypes identified in the UAMOCS framework demonstrate a high degree of trajectory, suggesting that AML patients with similar molecular biomarkers may also share common biological pathways.

### 3.5 Dysregulation of signaling pathways in UAMOCS

Intriguingly, pathway analysis in TCGA discovery dataset indicated universal dysregulation of different Hallmark pathways (Fig. 4D). Specifically, hallmark relevant to immune responses and tumorigenesis enriched in UAMOCS1. UAMOCS2 was specifically associated with pathways related to VEGFA‐mediated angiogenesis and hypoxia. Moreover, a unique activation of the *MYC* signaling pathway was observed in patients within the UAMOCS3 subtype, highlighting distinct molecular characteristics. Consistent activation of angiogenesis/hypoxia and *MYC* signaling pathways were observed in ihCAMs‐AML (Fig. 4E,F) and other public datasets (Fig. S6A–D).

### 3.6 High‐throughput drug sensitivity of different AML subtypes

**Fig. 5:**
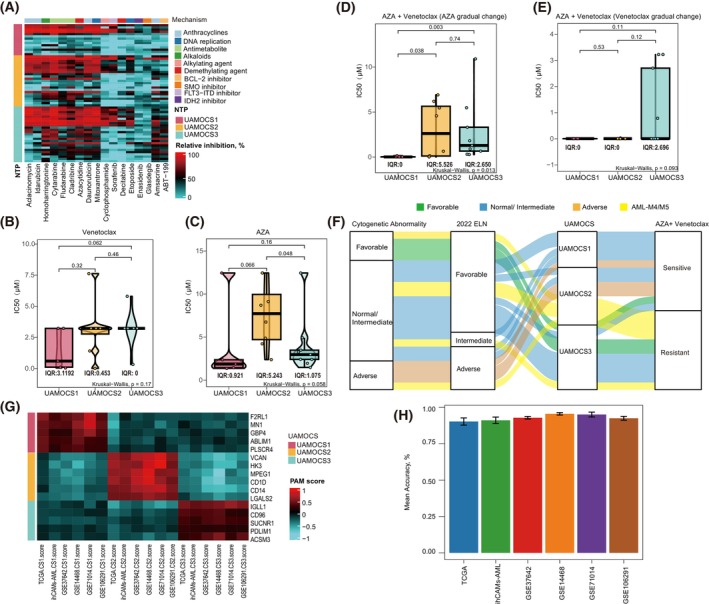
Drug sensitivity characteristics in ihCAMs‐AML cohort according to UAMOCS classification. (A) The heatmap illustrates the inhibition rates of 17 chemotherapeutic drugs at 100% peak plasma concentration, categorized according to UAMOCS subtypes. A higher inhibition rate indicates a more effective chemotherapeutic response. (B, C) The violin plots display the half‐maximal inhibitory concentration (IC_50_) of three UAMOCS subtypes following single drug *in vitro*, Kruskal–Wallis test. The box shows the interquartile range (IQR), with the upper and lower boundaries representing the 75th and 25th percentile IC_50_ value within each group, and the horizontal line indicating the median IC_50_ value. (B) was Venetoclax and (C) was AZA. (D, E) The violin plots display the IC_50_ of three UAMOCS subtypes following combined treatment with AZA and Venetoclax *in vitro*, Kruskal–Wallis test. The horizontal line represents the median IC_50_ value within each group. (D) shows the IC_50_ treated with a fixed concentration of Venetoclax and varying concentrations of AZA. (E) presents the IC_50_ following treatment with a fixed concentration of AZA and varying concentrations of Venetoclax. (F) The Sankey diagram illustrates a robust correlation between UAMOCS and cytogenetic abnormalities, the 2022 ELN guidelines, and the *ex vivo* drug responses to a fixed concentration of Venetoclax and varying concentrations of AZA within the ihCAMs‐AML cohort. (G) The heatmap displays the PAM scores for shaping the three AML subtypes, focusing on a refined 16‐gene panel for further clinical settings. (H) Accuracy Distribution of 16‐Gene Features by Random Forest Algorithm: The bar plot illustrates the classification accuracy distribution for 16 gene features, selected through the random forest algorithm over 10 000 iterations, across six independent cohorts. The error bars indicate SEM.

To assess the relationship between our UAMOCS subgroups and their response to various drugs, we assessed the inhibition rates of 17 chemotherapy drugs at 100% PPC *in vitro* across 62 ihCAMs‐AML cases. UAMOCS1 subgroup derived similar benefits from most chemotherapy drugs as the other two distinct subtypes (Fig. 5A and Table S8).

We further conducted IC_50_ determinations for 22 patients across seven different concentration gradients of AZA and Venetoclax. Although the hypomethylating agent AZA (average IC_50_: 2.365 μm vs. 7.6172 μm vs. 3.927 μm, *P* = 0.058) and the BCL‐2 inhibitor Venetoclax (average IC_50_: 1.438 μm vs. 3.145 μm vs. 3.188 μm, *P* = 0.17) alone did not improve outcomes for UAMOCS1 patients (Fig. 5B,C), different pattern emerged with combined therapy. When Venetoclax was used in conjunction with varying concentrations of AZA, significant differences in IC_50_ values were observed among the three subtypes (average IC_50_: 0.145 μm vs. 3.991 μm vs. 2.773 μm, *P* = 0.013), UAMOCS1 showing marked sensitivity compared to UAMOCS2 and UAMOCS3 in the combination drug screening (UAMOCS1 vs. UAMOCS2, *P* = 0.038; UAMOCS1 vs. UAMOCS3, *P* = 0.003) (Fig. 5D). Consequently, the sensitivity to combination therapy is predominantly associated with the concentration of Venetoclax, showing limited dependence on variations in AZA concentration. (Fig. 5E). UAMOCS 1–2 populations could benefit from BCL‐2 inhibitors (Venetoclax) combining with demethylating agents (AZA) (Fig. 5F), except monocyte‐type AML (M4/M5). In this term, UAMOCS may extend the capability of AML classification, populations that benefit from specific therapeutic regimens. This finding aligns with previous reports that monocytic cell types are prone to Venetoclax resistance.

### 3.7 A representative small size genes panel demonstrates high accuracy in subtype prediction

To accelerate translational medicine purpose in clinical setting, we develop a reduced small size panel for AML subtyping by PAM. Specifically, a total of 16 genes were narrowed down from 150 subtype dependency genes (Fig. 5G), including five genes from UAMOCS1, six genes for UAMOCS2, five genes for UAMOCS3 (Table S9). We tested the predictive classification ability of this 16‐gene panel among 1391 AML cases of six independent dataset using 10 000 repeated random forest classifiers. The accuracy distribution of all datasets was as follows: 0.919 (0.811–0.989) in TCGA, 0.908 (0.816–0.989) in ihCAMs‐AML, 0.9293 (0.892–0.960) in GSE37642, 0.9562 (0.922–0.983) in GSE14468, 0.952 (0.894–1) in GSE71014, and 0.924 (0.868–0.972) in GSE106291 (Fig. 5H). These findings suggest that the representative small panel could reach a comparative performance of full 150‐gene signature and was promising to be translated in clinical setting in the future.

### 3.8 Comparison between UAMOCS and established prognostic models

To evaluate the prognostic potential of UAMOCS, we referenced several well‐established AML prognostic signature models from prior studies (Table S9). We chose three of these previously developed models known for their effectiveness in survival prediction and calculated the risk panel scores for each UAMOCS subtype across all AML patients among six different cohorts.

**Fig. 6:**
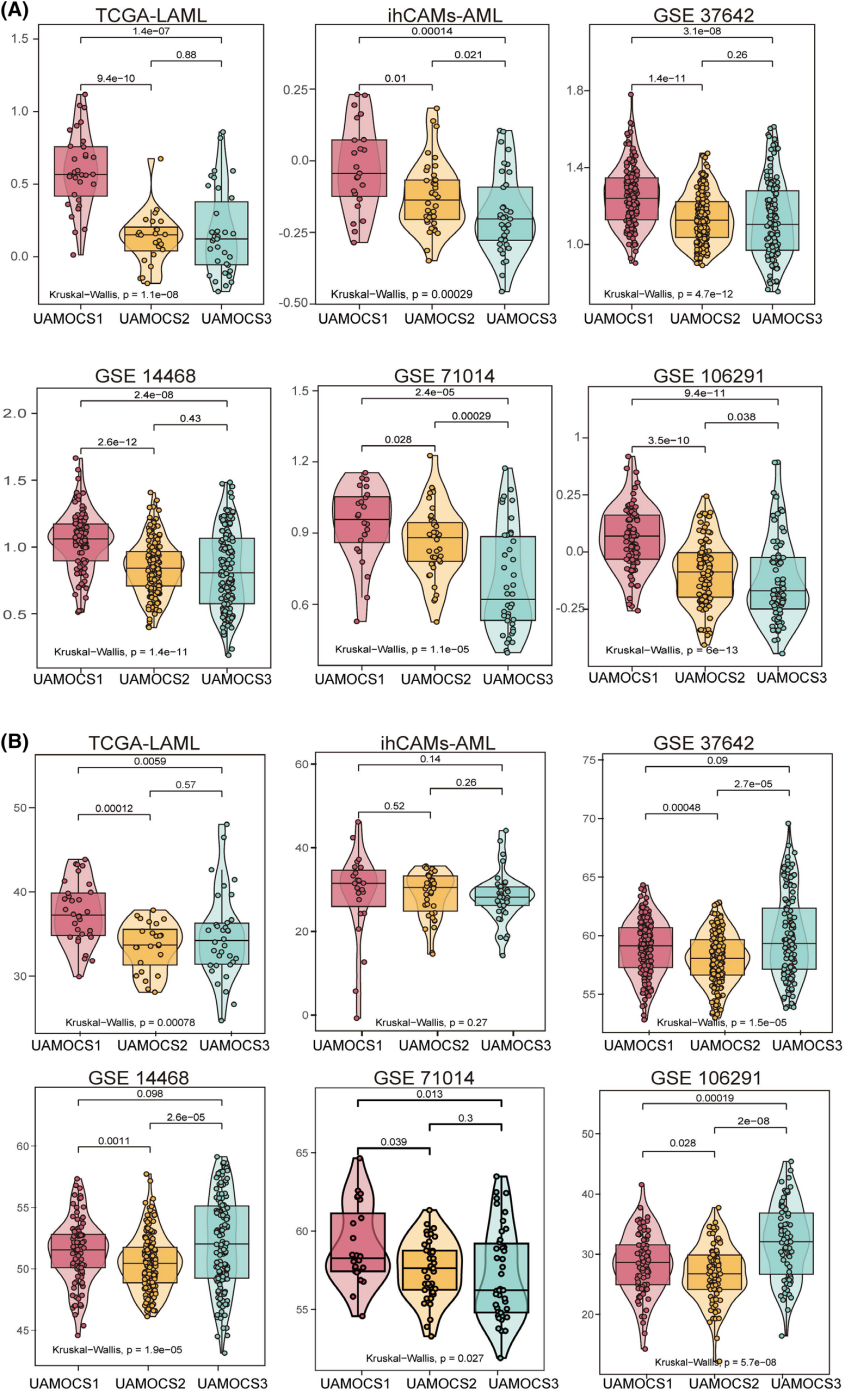
Characteristics of UAMOCS small panel and its comparison with previously established prognostic models. (A, B) The series of violin plots illustrates the distribution of a previously established prognostic score under UAMOCS classification across a variety of datasets, namely TCGA, iChAMs‐AML, GSE37642, GSE14468, GSE71014, and GSE106291. The horizontal axis represents the three subtypes of UAMOCS. The vertical axis represents the risk scores of patients with different subtypes. The three‐group difference was compared by Kruskal–Wallis test. The upper and lower boundaries in the box representing the 75th and 25th percentile risk scores, and the horizontal line indicating the median risk score. (A) This graph conveys the LSC 17 gene score [38] distribution classified by UAMOCS across six datasets. (B) This graph conveys the 24 gene score [39] distribution classified by UAMOCS across six datasets.

Our analysis has shown that prognostic risk signatures, derived from established classifiers, identify UAMOCS1 as high risk, UAMOCS2 as medium risk, and UAMOCS3 as low risk (LSC 17 gene score [38] in Fig. 6A, 24 gene score [39] in Fig. 6B). This outcome demonstrated high consistency between previously established prognostic models and the UAMOCS subtype distribution across the six external datasets. Importantly, this consistency underscores the value of our UAMOCS classifier as a reliable prognostic tool in the context of AML.

## 4 Discussion

Increasing evidence support the correlation between multi‐omics data and AML subtypes, spanning genomics, transcriptomics, proteomics, and epigenomics [10, 40, 41]. Integrated approach provides a comprehensive molecular landscape crucial for subtype distinction. Recent advances in AML subtyping, including leukemia cell hierarchy, clonal architecture, immune cell abundance, immune antigens, and cell differentiation, are vital to precise prognostic stratification and treatment strategy formulation [42, 43, 44, 45]. This study presents a framework for comprehensive AML subtyping.

Three UAMOCS subtypes were identified through multi‐omics integration. UAMOCS subtyping serves as a prognostic indicator that correlates with current genetic abnormalities and 2022 ELN and can be more precisely distinguished between adverse and intermediate‐risk group by using transcriptomic profiles. Additionally, they correlate with more comprehensive information, including immune status, biological implications, and clinical outcomes. This advancement offers an enriched understanding of the intricate interplay between the molecular features and biological behaviors of AML, significantly enhancing the precision of survival prognostication and the assessment of therapeutic outcomes.

Several well‐known signatures that influence leukemia prognosis were identified among the three AML subtypes, in accordance with ELN guidelines. AML‐MR mutations are associated with AML poor survival outcomes [46]. Characterized by hypermethylation, a high frequency of AML‐MR mutations, and unstable chromosomal changes, UAMOCS1 patients exhibit lower response rates to conventional therapies and poor prognosis. Conversely, the UAMOCS3 subgroup, containing majority of CEBPA‐bZip mutations, show the best prognosis [47, 48]. These clinical findings elucidate the connection between UAMOCS and the current standard AML risk consensus [2]. Additionally, UAMOCS genes strongly correlated with established prognostic models, such as the LSC 17 gene score [38] and 24 gene score [39], proving valuable to outcome forecasting. However, an expanding cohort is necessary to further validate these findings. This consistency underscores the prognostic value of our identified signatures, reinforcing their potential utility in a clinical setting.

Diverse immune status is closely associated with therapeutic responses in AML [49, 50]. We also observed that UAMOCS2 exhibited intermediate prognosis, which may be associated with the high infiltration of immunosuppressive cells, such as MDSCs and TITRs. Nevertheless, in our research, we discovered that the M4/M5 cluster in UAMOCS2 displayed more immune escape features through unsupervised clustering. The reason for the poor survival outcomes of monocyte‐derived AML may be its capacity to avoid T‐cell immunity [51]. Immature monocytic leukemia cells have been discovered to induce apoptosis in neighboring dysfunctional CD4/CD8+ T cells through the disruption of NADPH production, a process involved in the PARP‐1/PAR pathway [52]. Further investigation is needed to fully elucidate the underlying mechanisms. UAMOCS offers valuable insights beyond molecular genetics, and function as a comprehensive classifier system capable of clustering analogous immune phenotypes as well as comparable cell types.

Previous study has been demonstrated that the presence of monocytic subclone may be a contributing factor that AML patients do not respond to the BCL‐2 inhibitor‐based treatment regimen [53]. In our study, *ex vivo* drug sensitivity screening suggested that AML patients classified as relatively poor prognostic UAMOCS1‐2 subtypes might benefit from treatment with AZA and Venetoclax, but except FAB‐M4/M5. In this term, UAMOCS may also serve as potential biomarkers for identifying drug sensitivity, particularly to the AZA and Venetoclax regimen.

One of the most clinically relevant findings in this study is the unexpected sensitivity of UAMOCS1 patients to the combination of azacitidine and venetoclax. This subgroup, which primarily includes patients with myelodysplasia‐related mutations and chromosomal instability, typically has poor prognosis with conventional therapies. The fact that UAMOCS1 patients exhibit high sensitivity to azacitidine and venetoclax offers new insights into the therapeutic potential of this combination.

The rapid classification offered by UAMOCS enables clinicians to predict AML progression and response to treatment with greater accuracy. The creation of an R package named ‘UAMOCS’ extends the utility of this system by providing clinicians and researchers with an advanced computational tool designed specifically for analyzing and interpreting transcriptomic data within this framework.

While this study presents a robust multi‐omics framework for AML classification, certain limitations should be acknowledged. First, the relatively small sample sizes and scarcity of cases with matched RNA sequencing, methylation data, mutations, and comprehensive clinical profiles restrict the exploration of broader clinical implications. Additionally, the lack of other omics, such as proteomics and metabolomics data in our cohorts limits the ability to capture post‐translational modifications, protein interactions, and metabolic reprogramming, which are critical for a comprehensive understanding of AML biology. This limitation stems from the unavailability of matched proteomic and metabolomic datasets in both public repositories and our own cohort. Nevertheless, by integrating genomic, transcriptomic, and epigenomic data, our study represents one of the most comprehensive multi‐omics classifications for AML to date. Future research incorporating proteomics and metabolomics is essential to further refine subtype classifications and enhance prognostic predictions, potentially leading to a more detailed and clinically applicable stratification system for AML patients.

By acknowledging this limitation, we aim to provide a balanced view of the scope and applicability of our study. We believe our findings lay a strong foundation for further investigations that integrate additional omics data to enhance precision in AML classification and personalized treatment strategies.

## 5 Conclusions

Collectively, we have established the UAMOCS based on multi‐omics profiling. UAMOCS can redefine the characteristics of AML subtypes, predict prognosis comparable to ELN, and more effectively distinguish between intermediate‐ and high‐risk groups. It can also reflect the immune status of AML, guide the selection of treatment regimens, and predict patient responses to therapy.

## Conflict of interest

The authors declare no conflict of interest.

## Author contributions

YS designed the research, performed the bioinformatics and functional analysis, and wrote the manuscript; ZW and JH collected the clinical samples; KL, GZ, SW, YL, CZ, and DL provided patients samples and integrate the clinical data; MW, HW, and JW assisted in data interpretation; TC and YM designed and supervised the research.

## Peer review

The peer review history for this article is available at https://www.webofscience.com/api/gateway/wos/peer‐review/10.1002/1878‐0261.70000.

## Supporting information


**Fig. S1.** Detectable molecular and cytogenetic abnormality of three UAMOCS subtypes in TCGA‐LAML cohort.
**Fig. S2.** Characteristics of four public datasets as externally validated cohort through NTP algorithm.
**Fig. S3.** Validation of UAMOCS within the ihCAMs‐AML cohort.
**Fig. S4.** The prognostic value of UAMOCS across different datasets.
**Fig. S5.** Recognition of UAMOCS immune phenotype across four databases.
**Fig. S6.** Distinct upregulated signaling pathways characteristic of UAMOCS subtypes across four databases.
**Table S1.** The list of the demethylor gene probes.
**Table S2.** 267 hotspot mutations associated with malignant hematologic disorders.
**Table S3.** The 100% PPC of drug sensitivity for inhibition rate test *in vitro*.
**Table S4.** Independent test between UAMOCS subtype and 65 mutations.
**Table S5.** Subtype specific genes for UAMOCS.
**Table S6.** Clinical characteristics in ND ihCAMs‐AML cohort under UAMOCS.
**Table S7.** Clinical data regrading to CBF‐AML in ihCAMs‐AML cohort.
**Table S8.** The 100% PPC inhibition rate of three clusters under UAMOCS.
**Table S9.** The core genes in previous established prognostic models and our model.

## Data Availability

The raw sequence data were available in the Genome Sequence Archive in National Genomics Data Center built by China National Center for Bioinformation/Beijing Institute of Genomics, Chinese Academy of Sciences with accession number PRJCA018058 (HRA004996, HRA005008, and OMIX004472). The UAMOCS is available from https://github.com/songyang0106/UAMOCS as an R package. Other clinical data will be shared with qualified scientific and medical researchers, upon the researcher's request, as necessary for conducting legitimate research.
