# Supplementary material for: Classification of acute myeloid leukemia based on multi‐omics and prognosis prediction value
Source: Mol Oncol. 2025 Feb 10;19(6):1836–54. doi: 10.1002/1878-0261.70000 (PMC12161470; doi:10.1002/1878-0261.70000)
Supplement: Supplementary file 1 — Fig. S1. Detectable molecular and cytogenetic abnormality of three UAMOCS subtypes in TCGA‐LAML cohort. Fig. S2. Characteristics of four public datasets as externally validated cohort through NTP algorithm. Fig. S3. Validation of UAMOCS within the ihCAMs‐AML cohort. Fig. S4. The prognostic value of UAMOCS across different datasets. Fig. S5. Recognition of UAMOCS immune phenotype across four databases. Fig. S6. Distinct upregulated signaling pathways characteristic of UAMOCS subtypes across four databases. Table S1. The list of the demethylor gene probes. Table S2. 267 hotspot mutations associated with malignant hematologic disorders. Table S3. The 100% PPC of drug sensitivity for inhibition rate test in vitro. Table S4. Independent test between UAMOCS subtype and 65 mutations. Table S5. Subtype specific genes for UAMOCS. Table S6. Clinical characteristics in ND ihCAMs‐AML cohort under UAMOCS. Table S7. Clinical data regrading to CBF‐AML in ihCAMs‐AML cohort. Table S8. The 100% PPC inhibition rate of three clusters under UAMOCS. Table S9. The core genes in previous established prognostic models and our model. [file MOL2-19-1836-s001.pdf]

Supplementary materials

Classification of acute myeloid leukemia based on multi-omics and prognosis prediction value

Yang Song[a], Zhe Wang[a], Guangji Zhang[a], Jiangxue Hou[a], Kaiqi Liu[a], Shuning Wei[a], Yan Li[a], Chunlin Zhou[a], Dong Lin[a], Min Wang[a], Hui Wei[a], Jianxiang Wang[a], Tao Cheng[a*], Yingchang Mi[a*]

[a]State Key Laboratory of Experimental Hematology, National Clinical Research Center for Blood Diseases, Haihe Laboratory of Cell Ecosystem, Institute of Hematology & Blood Diseases Hospital, Chinese Academy of Medical Sciences & Peking Union Medical College, Tianjin 300020
Tianjin Institutes of Health Science, Tianjin 301600, China

[*]Address correspondence:

Yingchang Mi, State Key Laboratory of Experimental Hematology, National Clinical Research Center for Blood Diseases, Haihe Laboratory of Cell Ecosystem, Institute of Hematology & Blood Diseases Hospital, Chinese Academy of Medical Sciences & Peking Union Medical College, 288 Nanjing Road, Tianjin 300020, China. E-mail address: ychmi@ihcams.ac.cn

Tao Cheng, State Key Laboratory of Experimental Hematology, National Clinical Research Center for Blood Diseases, Haihe Laboratory of Cell Ecosystem, Institute of Hematology & Blood Diseases Hospital, Chinese Academy of Medical Sciences & Peking Union Medical College, 288 Nanjing Road, Tianjin 300020, China. E-mail address: chengtao@ihcams.ac.cn

Supplementary Guide

1. Supplementary Tables S1-S9---Page 3

2. Supplementary Figures S1-S6 ---Page 24

3. Supplementary Figure Legends--Page 31

4. Supplementary References--Page 33

Supplementary Table

Supplementary Table 1: The list of the demethylor gene probes

	CHR	Position	probe	CHR	Position	probe	CHR	Position
cg00037856	3	1.28E+08	cg09983285	10	1215399	cg22974210	3	1.23E+08
cg00087645	6	1.57E+08	cg10008947	17	73500739	cg22985929	19	6227536
cg00100114	7	1.02E+08	cg10012512	7	1.57E+08	cg22999786	15	34628769
cg00132141	10	69456307	cg10013455	10	1.34E+08	cg23007325	8	10924633
cg00185413	3	1.26E+08	cg10024799	12	2641381	cg23013977	19	36523056
cg00187327	22	28191781	cg10037894	1	1.72E+08	cg23014425	17	46648525
cg00193959	10	1.29E+08	cg10038168	12	1.24E+08	cg23019936	12	13903266
cg00202568	8	55528166	cg10064922	10	729846	cg23029597	12	1.23E+08
cg00245890	3	58574042	cg10085326	11	1.03E+08	cg23044178	11	12136405
cg00245896	4	8034100	cg10090414	8	1.42E+08	cg23079012	2	8343710
cg00277804	18	77549539	cg10090769	16	4740791	cg23092086	16	55909573
cg00281776	2	2.09E+08	cg10093265	13	25643742	cg23112821	14	1.01E+08
cg00285343	7	41733356	cg10113526	8	1.42E+08	cg23137088	5	1.35E+08
cg00288598	8	1.42E+08	cg10119001	1	1.53E+08	cg23167246	13	1.14E+08
cg00293660	6	55103999	cg10135708	3	48601117	cg23171972	8	1.43E+08
cg00294538	13	1.14E+08	cg10141261	10	1.26E+08	cg23186098	6	32056119
cg00299558	3	1.84E+08	cg10142874	2	11917623	cg23191354	7	25018565
cg00308065	12	1.24E+08	cg10149870	2	1.29E+08	cg23201907	14	1.05E+08
cg00310410	2	1.06E+08	cg10165952	17	58239449	cg23207876	19	15162821
cg00319595	16	75300861	cg10174170	17	33473881	cg23234999	15	23811205
cg00328597	1	2.37E+08	cg10176011	8	1905280	cg23235832	1	1.1E+08
cg00331027	5	80521615	cg10177030	20	47897207	cg23236370	5	33892705
cg00339331	19	33764550	cg10303653	6	32049516	cg23238315	7	1.56E+08
cg00341843	10	851265	cg10330187	16	88666383	cg23239150	4	5754217
cg00347938	11	69469113	cg10334750	8	1.01E+08	cg23244761	6	1.62E+08
cg00356499	4	3516065	cg10338112	16	30820353	cg23270529	1	2.44E+08
cg00367962	2	2.41E+08	cg10339295	14	1.02E+08	cg23275972	7	1.57E+08
cg00381745	7	99096450	cg10363337	8	1.45E+08	cg23296836	8	16870927
cg00383081	1	1.82E+08	cg10372421	16	1206807	cg23303108	8	23083578
cg00390784	6	2682491	cg10394757	16	66879807	cg23391288	13	49366203
cg00393348	6	1.59E+08	cg10436026	13	37453429	cg23411919	2	84517769
cg00411072	17	46660940	cg10438282	15	79471586	cg23419907	2	2.2E+08
cg00424286	6	1.67E+08	cg10442735	1	3062633	cg23445525	13	95055638
cg00436282	4	70861449	cg10473311	7	1.58E+08	cg23454797	7	27183990
cg00437411	15	59903968	cg10474881	6	1.14E+08	cg23493018	8	37309823
cg00443981	17	58499679	cg10476585	13	94499653	cg23497569	7	19417585
cg00461753	12	1.17E+08	cg10481072	7	92459517	cg23514400	15	75047329
cg00492070	3	1.08E+08	cg10493259	7	1.51E+08	cg23519637	7	1121738
cg00495708	2	1.37E+08	cg10499378	5	1.79E+08	cg23527387	2	1E+08
cg00499837	11	1.2E+08	cg10550166	15	34628731	cg23531049	15	42075277
cg00520042	17	17480931	cg10588362	11	64728445	cg23535322	14	95837929
cg00528492	6	32408863	cg10612997	2	11673928	cg23551720	17	46633726
cg00557959	13	1.13E+08	cg10621924	7	39171070	cg23556574	10	13981154
cg00562180	16	89638519	cg10622644	1	12538455	cg23590273	21	44105265

cg00565679	12	1.15E+08	cg10625247	2	2.21E+08	cg23597015	15	68932319
cg00576736	1	20030383	cg10645091	13	76445055	cg23606421	9	1.4E+08
cg00591333	17	79109910	cg10661002	7	25018503	cg23666945	3	32861150
cg00596184	16	69350052	cg10677351	19	1999003	cg23668057	17	76164111
cg00612985	2	1.01E+08	cg10708739	17	57465550	cg23698560	1	3822352
cg00620224	14	32030176	cg10753283	13	1.15E+08	cg23731272	15	67356838
cg00622170	4	2944594	cg10803218	5	1.78E+08	cg23755933	12	1.33E+08
cg00646731	17	46668733	cg10804344	2	4004633	cg23810282	5	43037519
cg00648582	12	1.33E+08	cg10822097	17	17480829	cg23813681	11	1.33E+08
cg00669330	2	2.43E+08	cg10825617	2	2.37E+08	cg23825231	17	45054460
cg00685614	16	89118838	cg10838380	10	1156113	cg23841288	7	1.58E+08
cg00701951	10	1.24E+08	cg10859192	12	54071672	cg23849826	15	39872186
cg00711496	19	50191497	cg10864794	5	78203170	cg23850913	6	15505452
cg00734931	6	9116428	cg10870892	11	70265971	cg23882164	2	9919440
cg00750630	14	51854261	cg10887937	12	1.12E+08	cg23886101	7	44438216
cg00774088	15	26109245	cg10906729	17	46682390	cg23887948	15	74494854
cg00787780	6	64151745	cg10941185	8	12988516	cg23891399	17	73824620
cg00793543	19	3650029	cg10959907	4	3374483	cg23969380	12	1.32E+08
cg00814985	13	1.15E+08	cg10976772	1	6390595	cg23972735	12	1.22E+08
cg00827581	19	10434088	cg10978034	6	33129988	cg23981702	1	1.16E+08
cg00827893	13	33162946	cg10994149	5	43037123	cg24000437	1	1.53E+08
cg00833441	7	98444410	cg11032640	2	1.51E+08	cg24020806	10	7448857
cg00835193	19	2291780	cg11056055	7	1.16E+08	cg24027068	11	1025969
cg00870269	15	58782688	cg11060532	17	46652399	cg24036292	11	70416269
cg00895196	6	22147182	cg11080430	17	16557885	cg24065451	11	1.28E+08
cg00908631	1	51433616	cg11099706	3	1.27E+08	cg24105081	12	1947479
cg00931644	13	77461368	cg11100640	15	23810861	cg24108508	1	1.79E+08
cg00933603	6	30883001	cg11104416	4	1202861	cg24118521	13	47472330
cg00935967	1	3822762	cg11117460	11	67982542	cg24134292	8	1.42E+08
cg00940514	20	60881152	cg11142556	17	80773629	cg24134897	11	859670
cg00950706	16	85262920	cg11149194	14	27245119	cg24150662	1	2949673
cg00966763	20	60472065	cg11185991	8	98194116	cg24154699	19	55141366
cg00986610	16	86554580	cg11217193	1	12538341	cg24177217	6	29702053
cg00989961	7	1.55E+08	cg11231069	2	2.4E+08	cg24177393	5	43037517
cg00994250	6	1.51E+08	cg11348106	17	75208807	cg24216893	2	1426674
cg00996827	1	2838805	cg11350447	5	87898610	cg24246472	22	48606007
cg01005322	20	25059984	cg11357542	2	66669737	cg24247086	17	1576724
cg01021169	22	30184971	cg11357670	13	1.14E+08	cg24249450	7	33894296
cg01026796	5	54602543	cg11377642	1	2.46E+08	cg24250902	1	2.3E+08
cg01054110	12	1.25E+08	cg11435167	19	1588449	cg24262654	4	1148456
cg01070760	18	23714009	cg11479811	2	2.41E+08	cg24267275	2	88958370
cg01077178	10	22608674	cg11520439	9	1.08E+08	cg24278165	8	23083551
cg01084500	1	2.45E+08	cg11530693	1	1.2E+08	cg24334174	1	22141341
cg01093088	1	1.53E+08	cg11532431	7	27169674	cg24336447	19	56674759
cg01140416	7	1293629	cg11549874	22	31202478	cg24361265	15	44068668
cg01146928	22	46455670	cg11557932	6	4806305	cg24361808	22	46477834
cg01148127	17	1478604	cg11580676	1	1.56E+08	cg24366429	17	77207424
cg01205267	13	47126300	cg11615509	4	77528588	cg24371534	1	3384929
cg01205935	13	99737585	cg11674448	1	41711766	cg24371954	13	1.11E+08

cg01206378	1	95698827	cg11682858	11	15822333	cg24375364	7	2755000
cg01207684	16	4103167	cg11684450	19	1272853	cg24412662	1	2866667
cg01214346	17	406501	cg11730703	14	1.05E+08	cg24424217	10	1.35E+08
cg01214458	22	28193996	cg11747081	13	76444823	cg24450303	8	1.44E+08
cg01228271	3	1.43E+08	cg11787218	6	312105	cg24471576	10	77118734
cg01254034	6	28543667	cg11827514	8	1.43E+08	cg24495350	15	72651198
cg01269299	10	1.12E+08	cg11838898	2	1.32E+08	cg24513014	1	7210826
cg01293277	12	10123440	cg11848015	17	79433150	cg24537237	11	1254204
cg01314743	7	1E+08	cg11858019	15	80246819	cg24537688	2	1.37E+08
cg01320579	17	75405842	cg11858305	18	23713595	cg24554151	11	93885823
cg01330448	13	1.14E+08	cg11864574	10	22635028	cg24574382	17	80333853
cg01335597	19	1944192	cg11902180	8	1780242	cg24601522	4	3287376
cg01364769	10	22607608	cg11920765	4	1.39E+08	cg24630778	12	1.33E+08
cg01389506	12	1.09E+08	cg11934832	18	45683841	cg24636368	17	46388447
cg01421140	1	57470853	cg11941630	20	1283388	cg24663971	14	1.06E+08
cg01425188	8	28479277	cg11979621	7	1.56E+08	cg24667758	18	24283696
cg01427750	12	1.32E+08	cg11983038	13	34251128	cg24756378	14	33401638
cg01433297	20	4151816	cg12015737	7	27184030	cg24756642	2	1597123
cg01433468	1	2.12E+08	cg12020778	8	1642577	cg24767968	17	46651945
cg01458510	2	1.1E+08	cg12114200	13	1.12E+08	cg24788483	10	1.15E+08
cg01479473	16	1524174	cg12117396	1	2438037	cg24794992	16	21689897
cg01481251	11	32912719	cg12121075	12	1.22E+08	cg24852565	13	44990174
cg01517500	2	10637974	cg12134349	8	1.42E+08	cg24860589	11	1.3E+08
cg01525839	16	88228776	cg12137473	8	1.45E+08	cg24881255	19	3772167
cg01527486	13	1.15E+08	cg12141457	10	1.26E+08	cg24921808	19	740313
cg01560554	19	55447414	cg12152540	10	28027746	cg24924502	17	79109777
cg01562813	3	13420826	cg12177944	3	1.29E+08	cg24925701	1	18553942
cg01569295	1	2.31E+08	cg12211091	19	2512999	cg24968629	22	46770644
cg01577029	12	1.33E+08	cg12220058	15	85203259	cg25020073	10	4925586
cg01582937	8	22411211	cg12226095	4	27057954	cg25035376	12	57943261
cg01588379	7	1.51E+08	cg12263469	11	69468863	cg25045942	6	33048291
cg01620540	13	47472064	cg12269111	2	1.69E+08	cg25065131	10	93100919
cg01621390	12	5195150	cg12294118	2	2.03E+08	cg25083922	8	1.42E+08
cg01623261	17	79377850	cg12294633	7	38356729	cg25106707	4	15341523
cg01634146	19	13127389	cg12306296	11	1107908	cg25113462	2	2.39E+08
cg01637125	14	77587383	cg12319143	10	1.35E+08	cg25135084	7	1.49E+08
cg01640684	17	80870529	cg12351768	13	1.13E+08	cg25161161	17	78190898
cg01642895	17	954410	cg12363903	5	1.35E+08	cg25239095	18	10589360
cg01646784	22	37976725	cg12382846	20	60892121	cg25254338	15	32158616
cg01662942	2	2.42E+08	cg12403162	10	1.16E+08	cg25261547	19	49363369
cg01667493	7	1.01E+08	cg12417466	3	35683819	cg25266888	11	2482594
cg01674777	8	1861037	cg12422199	3	1.01E+08	cg25279778	1	35250670
cg01724917	16	17563028	cg12426313	1	6552387	cg25282072	4	670347
cg01739831	16	89922539	cg12444166	4	8359458	cg25291653	20	19867145
cg01758575	16	28943288	cg12448285	1	2.46E+08	cg25294504	20	9491559
cg01763666	17	80159506	cg12458039	7	210075	cg25301756	4	1804106
cg01768926	18	32820085	cg12468255	1	2.02E+08	cg25310592	11	5174413
cg01775612	16	31484199	cg12472449	17	79022879	cg25314111	5	1.78E+08
cg01791669	12	1.09E+08	cg12486558	6	1.34E+08	cg25332717	2	1.06E+08

cg01799521	1	39305163
cg01803461	16	86604522
cg01817364	5	43037411
cg01820962	6	1.17E+08
cg01829163	16	87871160
cg01843999	5	1.37E+08
cg01854676	14	1.05E+08
cg01876548	5	1.24E+08
cg01882880	17	46623819
cg01883046	2	1.75E+08
cg01883662	3	1.96E+08
cg01898377	20	48960272
cg01898628	17	41836261
cg01904978	11	85847072
cg01941278	5	71895997
cg01946364	5	1.75E+08
cg01958934	19	45449099
cg01982835	20	34995278
cg01993552	19	46584209
cg01995393	19	8563628
cg01997884	2	2.25E+08
cg02000606	7	87103624
cg02010763	15	61044829
cg02030454	1	35250489
cg02043895	21	44835697
cg02048220	14	91141833
cg02070740	1	1.47E+08
cg02073465	15	59904009
cg02097532	3	55673955
cg02144924	22	45244265
cg02152631	8	29133647
cg02176678	2	2.2E+08
cg02185007	8	1.18E+08
cg02248749	6	32041191
cg02253236	19	1046996
cg02272278	6	1.58E+08
cg02281970	19	1271307
cg02282640	16	81743587
cg02293732	16	69304299
cg02295856	16	1758935
cg02319318	8	81639711
cg02339793	17	79225573
cg02364942	21	15588038
cg02386219	17	79030539
cg02409722	4	1.4E+08
cg02419835	5	60625717
cg02447879	17	79393682
cg02452491	13	74544655
cg02459469	12	1.16E+08
cg12486944	17	80159399
cg12501818	2	45224505
cg12560907	4	1.58E+08
cg12564034	17	1492127
cg12581741	10	1559249
cg12584394	19	2016427
cg12597309	12	1.24E+08
cg12604031	3	1.23E+08
cg12616941	10	22612797
cg12681784	17	79428750
cg12683120	20	50182196
cg12688965	8	1.45E+08
cg12721603	11	69482540
cg12732548	15	40631573
cg12744006	17	55083655
cg12762449	13	1.14E+08
cg12793733	4	47488620
cg12800047	11	1.07E+08
cg12806681	5	368394
cg12864235	5	27038782
cg12865888	8	1.41E+08
cg12872489	9	1.41E+08
cg12910797	17	46651722
cg12912293	1	1.51E+08
cg13001142	6	1.48E+08
cg13005613	9	1.37E+08
cg13038847	22	28073997
cg13081331	7	1308600
cg13083129	19	1311811
cg13114696	21	47808953
cg13133304	11	1862020
cg13172906	8	1.44E+08
cg13174084	2	84517877
cg13197560	13	1.14E+08
cg13207180	17	6558064
cg13280788	17	46652179
cg13293524	17	46651822
cg13305444	1	2.26E+08
cg13307782	5	77291146
cg13314778	3	70889448
cg13329242	11	1.17E+08
cg13341668	3	50359909
cg13342634	8	88972044
cg13351249	6	1.66E+08
cg13373343	1	95552972
cg13390332	17	78971641
cg13390484	9	80765924
cg13429424	15	22798849
cg13432945	1	59193388
cg25349729	1	2.15E+08
cg25414165	10	77542488
cg25420747	2	18112176
cg25436486	6	1.66E+08
cg25446191	3	1.7E+08
cg25458083	18	77645531
cg25499543	15	39871876
cg25517015	19	17584020
cg25540854	18	77404907
cg25553466	10	1.34E+08
cg25556035	19	13127873
cg25556225	15	31515750
cg25565203	5	56989227
cg25588348	14	76334455
cg25588389	7	2654420
cg25628461	4	687023
cg25670583	4	715865
cg25674938	19	1360981
cg25677261	15	1.02E+08
cg25712567	4	715872
cg25727671	7	27193351
cg25734089	19	46969919
cg25737218	19	944415
cg25754933	17	79429612
cg25758828	2	1.14E+08
cg25765464	19	30941858
cg25784136	22	21987106
cg25806190	2	2.33E+08
cg25822783	12	1.33E+08
cg25828445	12	7781288
cg25852019	2	2.2E+08
cg25929664	7	220654
cg25960090	5	1.27E+08
cg25965498	3	11746491
cg25972714	10	88024569
cg25983544	18	23713626
cg26020069	6	52382441
cg26020695	13	46355841
cg26033520	10	74004071
cg26040809	10	1505626
cg26072254	1	2.46E+08
cg26075259	7	1.57E+08
cg26081974	13	1.14E+08
cg26082814	13	1.14E+08
cg26101485	8	1.42E+08
cg26116326	5	1.73E+08
cg26130383	3	1.21E+08
cg26130533	4	1202700
cg26147480	10	1.26E+08

cg02480320	5	2135054	cg13435834	7	1123508	cg26156120	14	45579157
cg02483735	1	37051530	cg13443768	11	65084386	cg26172195	11	68972651
cg02485729	12	1.29E+08	cg13458803	3	1.19E+08	cg26224354	7	1096374
cg02487233	3	1.08E+08	cg13464117	7	1.57E+08	cg26234644	17	10634427
cg02518338	17	47929557	cg13469425	4	48175353	cg26267259	7	1.57E+08
cg02538833	1	9822207	cg13471521	1	12244930	cg26280578	20	61916036
cg02544002	3	1.29E+08	cg13506670	14	56045827	cg26337914	7	1.57E+08
cg02555772	16	1079316	cg13533424	9	1.38E+08	cg26348995	17	80345367
cg02567151	20	61915437	cg13572071	1	23707912	cg26385126	12	1.25E+08
cg02582848	1	2.24E+08	cg13583664	9	1.32E+08	cg26400491	8	1.45E+08
cg02607810	7	84671582	cg13605674	3	1.96E+08	cg26401541	6	91078974
cg02635875	8	1.44E+08	cg13640225	10	1.29E+08	cg26466027	3	45989744
cg02663352	11	35167041	cg13657981	7	1.35E+08	cg26479374	4	1243980
cg02715602	19	4544446	cg13698224	9	87309394	cg26490671	7	1.58E+08
cg02729344	16	49888237	cg13720395	7	75957902	cg26518660	1	79728608
cg02736280	17	1633745	cg13720710	10	1452970	cg26530341	8	23083353
cg02739280	11	20119229	cg13745832	17	15405942	cg26612409	5	160335
cg02744046	15	58782685	cg13751548	9	37005935	cg26657404	16	85684821
cg02748047	6	32373022	cg13756251	9	1.38E+08	cg26664254	10	1.32E+08
cg02761308	17	56071963	cg13761421	9	78652814	cg26677288	1	2.46E+08
cg02806739	17	80273242	cg13804476	13	1.14E+08	cg26695784	16	54101846
cg02816525	15	59024033	cg13817905	6	4381808	cg26706676	7	1.57E+08
cg02825728	21	31540204	cg13826666	9	1.37E+08	cg26707052	11	13250995
cg02831900	4	1.84E+08	cg13846675	8	53080905	cg26726230	17	79225857
cg02836478	17	46652501	cg13871843	1	9887984	cg26730619	19	57743416
cg02913194	6	1.44E+08	cg13872898	2	1.21E+08	cg26740109	18	77585428
cg02965295	2	1.98E+08	cg13878677	3	13060915	cg26754761	2	1.77E+08
cg02987249	19	6750327	cg13880868	9	1.31E+08	cg26764761	16	87682142
cg03062717	19	35940483	cg13889415	9	1.17E+08	cg26772538	8	1824939
cg03080336	4	1.86E+08	cg13911501	8	1.41E+08	cg26797898	12	1.31E+08
cg03089940	8	21767146	cg13911959	10	1505654	cg26806713	6	1.7E+08
cg03099780	13	97864475	cg13922442	7	38312366	cg26840970	16	71523432
cg03144619	1	2.3E+08	cg13936972	19	10221907	cg26848442	18	67624264
cg03173502	6	15505345	cg13969327	7	1.28E+08	cg26865747	6	28601377
cg03233624	17	55740464	cg14013695	7	27184176	cg26869506	10	1.34E+08
cg03233876	19	575412	cg14040931	9	36249297	cg26872907	6	1.62E+08
cg03256597	19	1280967	cg14050824	9	1.41E+08	cg26873880	6	1.6E+08
cg03256938	1	2983926	cg14058329	7	27183946	cg26879349	15	26109215
cg03258927	1	1.15E+08	cg14072016	10	384652	cg26883434	5	1.11E+08
cg03284308	16	81039325	cg14072140	1	98031839	cg26895622	10	1.35E+08
cg03317245	17	3600514	cg14085952	9	1.39E+08	cg26902127	16	88513285
cg03326410	6	64151739	cg14157549	6	1.68E+08	cg26902279	5	1.35E+08
cg03328299	15	74281983	cg14218053	6	32049376	cg26916576	14	91700557
cg03365403	11	421691	cg14225665	14	1.02E+08	cg26925644	11	1.18E+08
cg03366574	7	2764599	cg14288049	9	1.29E+08	cg26953469	10	31872296
cg03393445	8	19249545	cg14291900	14	23294368	cg26982321	6	1.38E+08
cg03399574	13	19759420	cg14332367	17	78058721	cg26986438	17	80542006
cg03419014	21	38362742	cg14360448	9	1.4E+08	cg26994885	12	77260534
cg03442712	15	86292964	cg14387312	4	53704546	cg26996656	16	880371

cg03458695	10	78163703	cg14392283	8	1.44E+08	cg27022324	5	170081
cg03470671	11	45115584	cg14425733	22	46451214	cg27055601	13	1.14E+08
cg03475293	6	7051303	cg14447608	9	1.34E+08	cg27059698	17	17625521
cg03504039	2	2.39E+08	cg14469972	9	1.41E+08	cg27064178	22	50178220
cg03509771	8	48091864	cg14482811	9	1.4E+08	cg27087809	15	78527410
cg03512414	20	19915874	cg14486098	17	79267785	cg27109748	7	2150016
cg03532673	7	1.03E+08	cg14507445	22	49886340	cg27140633	11	63685905
cg03534481	21	34439545	cg14534144	13	1.14E+08	cg27154418	19	55526208
cg03534846	19	1999322	cg14599823	5	77973731	cg27162122	4	1.84E+08
cg03538383	12	1.1E+08	cg14618803	8	48091801	cg27174787	19	16178240
cg03556243	3	1.14E+08	cg14630748	6	32014484	cg27195956	3	32861910
cg03564727	19	49577206	cg14633252	9	37964247	cg27214856	22	46473343
cg03565777	12	1.25E+08	cg14651210	1	1.83E+08	cg27217555	4	2943901
cg03579045	8	11579013	cg14679558	13	21652153	cg27221631	4	77703381
cg03586793	14	1.04E+08	cg14711743	5	79514577	cg27261733	11	1891872
cg03602500	19	55047721	cg14715697	17	77127487	cg27268708	4	11700861
cg03608093	11	691457	cg14773228	2	1.22E+08	cg27300804	13	1.14E+08
cg03625953	17	1975218	cg14795528	7	1.57E+08	cg27312312	14	56046001
cg03666350	2	46662132	cg14845609	13	1.13E+08	cg27326318	11	70268987
cg03691722	18	7011268	cg14886629	3	10333655	cg27336261	10	1975562
cg03716032	8	1.42E+08	cg15014975	1	25257547	cg27337124	10	1.32E+08
cg03731458	10	11246763	cg15018934	6	29689744	cg27359668	13	1.14E+08
cg03748603	19	2494443	cg15048806	6	32049373	cg27377632	19	677895
cg03755052	16	57563395	cg15059065	19	17354961	cg27390253	19	11657100
cg03758367	12	54867542	cg15079934	11	1892307	cg27399125	6	1.5E+08
cg03791955	15	65024147	cg15090899	6	1.67E+08	cg27408238	7	1.5E+08
cg03797504	16	46660546	cg15098643	19	30365441	cg27409514	19	1169138
cg03834411	16	87682036	cg15108537	10	1.12E+08	cg27418402	3	64008161
cg03875996	4	1.59E+08	cg15127879	8	69917477	cg27422467	11	69482104
cg03907363	19	51326682	cg15136129	17	39646338	cg27422857	2	1.06E+08
cg03963853	16	4732369	cg15139179	10	1.32E+08	cg27438152	15	31515761
cg03974193	2	1.02E+08	cg15142819	6	1.58E+08	cg27452278	8	1.02E+08
cg03980224	5	34686875	cg15154628	7	76871793	cg27477250	19	12841735
cg04003327	2	2.39E+08	cg15188939	15	72809154	cg27484582	7	2518723
cg04024417	7	96294348	cg15202552	1	1.58E+08	cg27506210	22	32150725
cg04036306	16	89005054	cg15212455	7	39170539	cg27554954	15	60691595
cg04051927	2	4183531	cg15226226	13	76060574	cg27558541	1	1.1E+08
cg04055693	8	52308227	cg15230985	17	78753887	cg27565966	16	28943198
cg04064254	21	47409570	cg15231205	21	44573854	cg27566947	15	34628815
cg04067249	6	53330099	cg15318546	12	1.33E+08	cg27593250	6	30859984
cg04100337	17	71336777	cg15346842	2	59477066	cg27601906	8	95271635
cg04100395	17	6984187	cg15391531	18	74691423	cg27608981	7	1.02E+08
cg04105726	1	1601205	cg15395971	1	95010070	cg27630153	16	88845038
cg04115740	17	6690948	cg15401862	12	70824537	cg27634295	12	8043600
cg04117801	17	46651867	cg15411840	6	1.1E+08	cg27640020	19	19002253
cg04118102	17	15824118	cg15462970	10	1444586	cg27655168	17	39059398
cg04118124	1	43231249	cg15463284	11	18477534	cg27661460	16	88844969
cg04141129	12	32530696	cg15465279	14	1.05E+08	cg17562226	6	29394654
cg04143909	1	2.34E+08	cg15467834	21	44574022	cg17579089	3	47269695

cg04153130	7	4456396	cg15473218	19	18109045	cg17588094	8	1.29E+08
cg04154424	5	17204524	cg15491102	13	51027597	cg17601191	13	31264764
cg04172115	6	32053728	cg15574442	1	2437658	cg17611647	17	75260857
cg04191427	17	78833259	cg15604241	11	35941235	cg17614801	17	77128546
cg04191989	7	630914	cg15630071	3	32861318	cg17629148	17	47113596
cg04203702	4	11369349	cg15642307	6	32061084	cg17631184	11	70266172
cg04239078	1	2.1E+08	cg15651928	20	32290811	cg17702388	19	16466480
cg04248279	17	184833	cg15674899	5	1.51E+08	cg17720259	10	1.32E+08
cg04268624	5	43037285	cg15695155	12	1.22E+08	cg17740093	1	22916803
cg04275506	1	12445337	cg15700989	17	79442098	cg17760049	7	4774448
cg04339462	14	1E+08	cg15742700	8	11350853	cg17787876	1	1.56E+08
cg04357789	6	14933411	cg15756319	13	1.13E+08	cg17818613	2	97527586
cg04364311	3	1.01E+08	cg15813011	12	1.32E+08	cg17820022	1	2425860
cg04370829	17	406249	cg15819225	1	3583333	cg17862447	2	23633680
cg04414509	3	1.69E+08	cg15885274	4	6891202	cg17869988	1	1601380
cg04446647	13	24878887	cg15901783	13	77461426	cg17873456	13	1.07E+08
cg04450876	12	54866945	cg15902830	1	3387913	cg17890940	7	1782346
cg04468081	3	1.83E+08	cg15979932	17	55980107	cg17929273	22	37967569
cg04503267	1	32742037	cg15997518	1	81814874	cg17955329	12	1.14E+08
cg04505348	20	3651823	cg16005592	5	964654	cg17960164	8	1.45E+08
cg04507915	3	32861407	cg16018154	17	78863570	cg17970299	12	54772804
cg04521543	2	669505	cg16034168	1	6336711	cg17999280	6	1.62E+08
cg04535902	1	92947332	cg16043345	4	88571325	cg18011760	2	19320928
cg04566512	22	46457588	cg16050468	14	1.05E+08	cg18029167	8	1.11E+08
cg04605697	10	53149243	cg16057915	1	10690564	cg18044967	11	2334942
cg04617640	21	46874243	cg16067628	17	79109808	cg18072388	19	10433761
cg04633141	7	1588319	cg16084133	22	28074146	cg18076842	11	93262607
cg04637264	6	1.12E+08	cg16104276	1	44880317	cg18085660	4	1202898
cg04647918	11	67383862	cg16139867	4	1.77E+08	cg18089380	3	55232663
cg04651548	8	1.43E+08	cg16184827	2	98961824	cg18105529	17	7643190
cg04664179	19	40913800	cg16212074	19	4170115	cg18127922	17	46672255
cg04717045	11	69468789	cg16257533	11	1.32E+08	cg18146737	1	92946700
cg04726535	12	1.33E+08	cg16284674	10	1.35E+08	cg18174881	14	45723353
cg04747693	16	88111362	cg16286735	17	45371778	cg18200810	13	47472200
cg04764839	10	18708076	cg16307866	4	7129517	cg18205883	10	821718
cg04787417	3	77146138	cg16338011	3	1.37E+08	cg18209359	17	80159595
cg04804543	8	1.42E+08	cg16356013	5	1.35E+08	cg18240543	10	99631008
cg04817870	17	79963651	cg16364066	10	6821184	cg18252648	3	1.5E+08
cg04819180	17	80829157	cg16365424	1	2.35E+08	cg18281910	1	2.37E+08
cg04843555	5	17360014	cg16366355	22	46459327	cg18286474	5	1.24E+08
cg04857395	4	3516637	cg16386293	1	1.5E+08	cg18313416	3	13420961
cg04892643	7	18906433	cg16399632	4	1244006	cg18320854	17	79001543
cg04908088	13	20925063	cg16417876	15	26109261	cg18384588	22	46463747
cg04918770	6	18053826	cg16446346	13	1.15E+08	cg18425570	17	39597980
cg04928457	4	1.87E+08	cg16477611	16	50896074	cg18476766	2	2.41E+08
cg04964883	6	1410436	cg16490015	14	1.05E+08	cg18484299	2	1.72E+08
cg04997124	14	1.01E+08	cg16510128	5	42757815	cg18485530	13	59547037
cg05000761	18	10589150	cg16524240	15	93316187	cg18515868	4	1304836
cg05017994	5	964562	cg16536610	14	65704960	cg18555698	11	12699172

cg05024916	6	1.71E+08	cg16538390	10	1.35E+08	cg18583565	10	1.32E+08
cg05025612	2	2.07E+08	cg16554447	6	54058452	cg18585107	11	64138839
cg05031187	7	73820613	cg16564828	13	1.14E+08	cg18586212	5	1.46E+08
cg05033389	1	15170397	cg16571642	7	1.58E+08	cg18612209	10	1.35E+08
cg05041061	17	79426049	cg16624069	13	99630210	cg18664013	7	1.58E+08
cg05061886	22	28192794	cg16639692	5	43037666	cg18666748	19	733352
cg05085169	20	61443751	cg16694785	1	3163710	cg18683606	7	1E+08
cg05090785	7	32099501	cg16699818	16	48479333	cg18684142	17	46682394
cg05132782	7	1.5E+08	cg16704797	2	23838416	cg18704979	3	47054668
cg05137975	6	99786314	cg16711084	6	1.7E+08	cg18722086	3	1.13E+08
cg05162523	1	25240938	cg16721321	4	1202696	cg18741958	3	1.25E+08
cg05168012	2	1.03E+08	cg16726039	12	1.33E+08	cg18749404	17	76975944
cg05169099	5	481640	cg16755833	17	80866063	cg18805164	19	36265700
cg05171197	2	2.4E+08	cg16765600	18	10589169	cg18863119	14	1.02E+08
cg05177729	11	69469349	cg16767590	4	715950	cg18915156	17	16419585
cg05202616	12	1.11E+08	cg16784985	21	44105474	cg18946602	1	61549982
cg05240760	2	1.99E+08	cg16786640	4	3485263	cg18950481	1	2.47E+08
cg05255168	16	1518657	cg16851758	2	84517782	cg18970151	13	1.01E+08
cg05259891	2	1.03E+08	cg16887070	6	3324467	cg19007167	2	2.43E+08
cg05267427	4	1.47E+08	cg16899088	12	10827583	cg19028462	13	1.14E+08
cg05283685	11	92001577	cg16908083	19	8563755	cg19041419	4	55527240
cg05305278	15	45803025	cg16924102	4	20044588	cg19045002	17	79380585
cg05372727	13	1.07E+08	cg16972240	19	4544574	cg19053454	5	1.8E+08
cg05373251	17	79451985	cg17002328	14	91751773	cg19071490	10	1.35E+08
cg05383958	3	43914187	cg17022362	20	62084833	cg19091930	5	191127
cg05399692	13	46190198	cg17031787	6	32049235	cg19101625	13	95369785
cg05399703	15	78527275	cg17064870	14	1.02E+08	cg19103950	7	1.05E+08
cg05411165	6	27466334	cg17097293	19	3655825	cg19106326	8	38575349
cg05415840	8	1.44E+08	cg17107691	19	11289376	cg19125584	7	601828
cg05430997	13	1.1E+08	cg17168093	2	85531486	cg19137417	17	79362935
cg05433111	16	28943232	cg17181543	1	77746571	cg19148731	2	74780229
cg05445244	4	1.64E+08	cg17184165	3	53855929	cg19190593	6	6588737
cg05445326	3	1.96E+08	cg17192381	18	60792598	cg19207017	10	1.04E+08
cg05457730	16	86795490	cg17192599	7	1.52E+08	cg19248395	1	64660725
cg05483571	17	77901224	cg17227257	4	1202694	cg19258177	1	95056619
cg05492735	7	1.04E+08	cg17250537	8	1.45E+08	cg19299265	10	1975631
cg05497253	5	77830465	cg17283266	11	1.12E+08	cg19300549	6	54200888
cg05528899	17	57120	cg17342469	22	46473074	cg19311375	2	46542361
cg05555337	17	46669113	cg17353893	7	73753326	cg19366753	7	78030012
cg05563033	13	1.01E+08	cg17413460	12	96367302	cg19393677	15	67841407
cg05612654	6	2375895	cg17438457	1	53094893	cg19413350	8	57351067
cg05615150	3	35683815	cg17461789	5	1.23E+08	cg19449565	2	2.4E+08
cg05695015	11	1.2E+08	cg17463698	15	83477712	cg19473656	14	23623663
cg05697866	1	2437200	cg17481703	13	76444831	cg19473989	4	7912384
cg05703996	16	335830	cg17485141	2	42566556	cg19474833	17	46622899
cg05707492	17	78833484	cg17500632	11	64694445	cg19477247	3	1.14E+08
cg05714496	10	1.34E+08	cg17519749	2	2111648	cg19495444	11	44291407
cg05750078	10	1567746	cg17527195	13	49403484	cg19515398	8	1.42E+08
cg05776053	2	74358815	cg08503681	5	1.46E+08	cg19531230	4	6072199

cg05803790	9	80765939	cg08509907	16	31477327	cg19560831	14	1.02E+08
cg05813818	16	875966	cg08511757	16	89342065	cg19572051	10	1.29E+08
cg05828497	4	1.74E+08	cg08541649	7	24826962	cg19579652	10	433089
cg05830220	16	87757033	cg08549011	5	1.33E+08	cg19586199	19	14225172
cg05830842	8	1.21E+08	cg08554603	7	1.21E+08	cg19589358	14	1.03E+08
cg05832823	13	75859662	cg08558886	2	1.51E+08	cg19618279	17	40715228
cg05887047	13	1.14E+08	cg08597832	8	1.44E+08	cg19620758	11	1.03E+08
cg05892024	7	553412	cg08620329	8	67454546	cg19628739	22	44317517
cg05892606	5	1.17E+08	cg08655953	1	2425888	cg19629631	7	2060116
cg05898618	11	2555576	cg08657449	8	1.05E+08	cg19631815	12	1.14E+08
cg05899869	6	3053528	cg08669954	16	1582105	cg19685567	7	1.56E+08
cg05900955	13	1.15E+08	cg08694574	16	86011615	cg19688203	14	1.02E+08
cg05903720	14	1.05E+08	cg08697732	17	81014091	cg19727396	1	2.3E+08
cg05927579	20	43378669	cg08718050	11	70563677	cg19764489	11	2730616
cg05973555	2	84114418	cg08754268	2	1.74E+08	cg19771748	12	1.17E+08
cg05987787	6	1.58E+08	cg08801479	6	32165200	cg19773937	1	60216631
cg06025774	5	92498282	cg08801887	11	67811194	cg19788371	8	1.43E+08
cg06039988	11	65158399	cg08819008	11	1.11E+08	cg19795793	2	2.07E+08
cg06047886	8	1947038	cg08845973	16	89592071	cg19805377	17	1576616
cg06052090	7	55100608	cg08855903	16	2121747	cg19824242	2	1.05E+08
cg06070002	1	45986120	cg08869722	1	76891398	cg19832347	13	24887606
cg06083412	8	71228213	cg08911692	7	1.57E+08	cg19856241	4	1.48E+08
cg06102242	16	84336383	cg08915171	2	1.05E+08	cg19866939	13	1.15E+08
cg06123807	7	2761760	cg08917664	1	1.47E+08	cg19904322	21	45249837
cg06155303	1	29460817	cg08920252	16	85262820	cg19910327	10	95723024
cg06167730	8	991533	cg08936645	4	37910273	cg19919989	5	66492464
cg06186155	17	46648582	cg08976646	5	77299683	cg19958593	14	87265617
cg06216650	2	558183	cg08985968	13	1.01E+08	cg19972648	10	79110632
cg06217314	6	28543693	cg08993681	13	1.07E+08	cg19998289	12	1.22E+08
cg06223834	16	4103161	cg09006514	2	1.28E+08	cg20005742	14	1.03E+08
cg06224587	4	6540449	cg09042966	13	1.04E+08	cg20032056	11	1.3E+08
cg06285909	16	69098945	cg09069072	1	15482753	cg20056593	12	1.33E+08
cg06286796	3	64008041	cg09075515	8	56192721	cg20063728	7	553423
cg06301399	6	29634495	cg09102260	13	31294292	cg20084184	4	1148524
cg06320175	1	1.72E+08	cg09137125	10	7486981	cg20095851	12	6486701
cg06330323	16	2132315	cg09141075	8	884716	cg20102045	13	1.1E+08
cg06338710	1	92946187	cg09156207	6	28058724	cg20116929	6	32049825
cg06345027	10	74695298	cg09172973	16	31091608	cg20119891	11	27017005
cg06349450	16	85867170	cg09194778	6	792749	cg20152430	17	46641504
cg06361984	16	15787940	cg09221482	6	1.62E+08	cg20159095	7	1.57E+08
cg06367607	6	1.07E+08	cg09230756	16	86774095	cg20187173	3	1.77E+08
cg06376992	15	79296424	cg09233791	6	1.67E+08	cg20193057	13	30079441
cg06436667	17	78058704	cg09236152	7	1.57E+08	cg20210376	11	65315466
cg06440474	1	1118965	cg09241368	12	78851733	cg20223392	8	328499
cg06446432	1	77307268	cg09244277	2	12538247	cg20225246	19	2200020
cg06454084	19	47158242	cg09251891	19	11374363	cg20229025	11	971902
cg06466348	16	50337922	cg09261793	10	1215440	cg20235117	3	10368030
cg06473363	6	31631797	cg09263316	16	2345275	cg20250162	19	10333164
cg06489615	16	1518592	cg09285851	6	10099656	cg20298668	2	2.4E+08

cg06505619	16	698072	cg09317508	1	5896560	cg20300129	6	35058181
cg06568346	20	44352696	cg09337069	14	1.02E+08	cg20311002	1	2.45E+08
cg06585734	3	46037942	cg09352338	11	70266139	cg20343063	11	57089744
cg06613738	16	15596423	cg09375756	11	692963	cg20344388	21	46829943
cg06638147	12	1.04E+08	cg09408571	1	1.01E+08	cg20346503	2	1.29E+08
cg06646780	6	1.3E+08	cg09427273	10	11077625	cg20359042	13	99135583
cg06651450	3	32860909	cg09461851	16	921161	cg20405742	6	16933816
cg06671298	17	79426159	cg09484541	5	74161187	cg20451050	12	92955405
cg06693983	19	55889216	cg09489844	17	79880647	cg20476021	11	1.23E+08
cg06703469	11	22301379	cg09520857	2	2.42E+08	cg20541752	13	54821786
cg06710785	10	83683829	cg09539496	8	20164433	cg20603222	7	1096387
cg06718533	16	86012468	cg09552399	16	31091326	cg20647257	7	601713
cg06741896	11	69468337	cg09556515	16	85470485	cg20655119	4	40425917
cg06741996	21	45149373	cg09567048	2	46456880	cg20683681	12	1.33E+08
cg06755452	5	966019	cg09574009	13	55211764	cg20722088	12	89742886
cg06790305	21	32412191	cg09588074	13	1.12E+08	cg20773033	22	39635187
cg06806080	3	1.08E+08	cg09595384	11	1.34E+08	cg20792436	17	79972570
cg06818159	4	16628713	cg09599228	4	1.59E+08	cg20806725	13	76444850
cg06819373	7	1.57E+08	cg09628822	15	60803663	cg20817131	7	27184167
cg06823108	18	59221320	cg09632163	16	2339292	cg20829550	12	93129447
cg06826975	2	98276287	cg09639964	17	909300	cg20830867	3	1.82E+08
cg06871344	17	452707	cg09654300	3	42132991	cg20842915	7	39665132
cg06894710	10	78142662	cg09654669	8	57350985	cg20873801	11	1.3E+08
cg06911238	1	46859671	cg09668627	1	1371844	cg20892919	12	65927379
cg07000686	10	47134299	cg09703963	11	616879	cg20894963	12	9885564
cg07002540	2	85281128	cg09718251	10	1132730	cg20918393	20	19867136
cg07012128	8	1448361	cg09734162	16	557929	cg20928950	13	1.14E+08
cg07031070	6	28543670	cg09734418	2	9549336	cg20936529	18	72912760
cg07045089	1	3620218	cg09750210	6	52985055	cg20942910	1	51812011
cg07058998	18	52625365	cg09768257	8	11628346	cg20952434	19	2016402
cg07068570	4	88571333	cg09775648	2	1.9E+08	cg20960181	1	86094825
cg07086353	11	64257660	cg09796089	14	1.05E+08	cg20973476	12	1.33E+08
cg07103673	11	7092719	cg09804496	7	72850442	cg20976787	6	43064460
cg07108927	18	24283669	cg09831562	3	1.81E+08	cg20978460	7	2644443
cg07122805	17	67754138	cg09836921	16	1582320	cg20979921	22	37584018
cg07144720	3	95038960	cg09857273	8	95230984	cg21024234	15	51964706
cg07165610	3	39231277	cg09860353	2	1.18E+08	cg21036605	6	79792291
cg07185119	2	31459843	cg09865015	4	1202509	cg21078330	2	1.34E+08
cg07224469	4	716282	cg09869226	11	70253427	cg21093005	17	80541915
cg07240413	10	8145066	cg09879253	17	45935359	cg21117808	1	9887936
cg07243548	1	1.08E+08	cg09892262	21	31655654	cg21145248	5	1.77E+08
cg07277756	18	10589357	cg09906991	11	1248676	cg21156386	11	692983
cg07280985	19	55549414	cg09907068	2	1.97E+08	cg21164005	14	1.03E+08
cg07296926	2	2.24E+08	cg09918751	15	1.01E+08	cg21167761	11	77903617
cg07306331	6	32049354	cg09935388	1	92947588	cg21182196	14	95786801
cg07350977	5	27038836	cg09936008	16	3188556	cg21197224	17	56082780
cg07354679	16	1206844	cg09959150	8	709624	cg21211645	3	45590039
cg07357157	1	10690588	cg09975715	17	8379050	cg21221786	9	1.27E+08
cg07366506	10	1.32E+08	cg09981464	16	87441659	cg21231141	14	1.03E+08

cg07385694	12	1.33E+08	cg08131184	19	33764378	cg21235678	7	1130967
cg07444408	4	1.41E+08	cg08148801	8	12992570	cg21239227	2	2111623
cg07470639	17	20839291	cg08152198	21	46087026	cg21336434	7	54271282
cg07470694	15	26109249	cg08162124	13	1.13E+08	cg21368566	2	2.37E+08
cg07479615	11	1470155	cg08172479	16	16951653	cg21393885	17	79313913
cg07509992	4	3742224	cg08183074	13	1.13E+08	cg21398111	10	75528810
cg07536737	2	98630091	cg08199758	16	3062426	cg21467935	6	31781906
cg07538160	6	30951394	cg08204867	16	10208426	cg21468274	17	7339835
cg07545858	16	88482626	cg08216099	2	1725761	cg21484834	20	62168579
cg07546433	8	1.02E+08	cg08259168	1	78509494	cg21488876	19	5250376
cg07554408	14	95837801	cg08259506	10	11937255	cg21526778	1	2.44E+08
cg07582923	14	1.05E+08	cg08266839	11	691488	cg21528399	4	24771586
cg07621803	18	76674796	cg08287334	19	1854633	cg21591938	12	29195033
cg07622957	16	1503662	cg08297640	4	3516758	cg21640654	2	2.34E+08
cg07633835	10	5938186	cg08318371	2	1.63E+08	cg21642988	15	78558406
cg07642499	11	69957602	cg08329113	16	70771142	cg21655923	2	74647813
cg07654864	16	19183628	cg08343075	16	90077951	cg21665744	7	39171113
cg07676349	19	5242136	cg08363339	20	57618487	cg21672992	15	86584249
cg07676920	3	12447615	cg08377000	4	90033921	cg21690793	6	792658
cg07698804	17	80829309	cg08381046	5	1.12E+08	cg21785245	1	77813387
cg07716089	17	17480461	cg08396193	7	27193709	cg21818891	11	35310917
cg07716927	7	4911224	cg08401628	16	85433676	cg21846949	2	33500889
cg07719172	12	1.1E+08	cg08402963	13	99135625	cg21864868	17	46673002
cg07722360	20	17691454	cg08417857	13	1.02E+08	cg21886042	13	1.12E+08
cg07783477	2	2.18E+08	cg08425543	2	50388775	cg22053945	17	46651360
cg07803375	7	811206	cg08439439	6	1.49E+08	cg22160073	11	5653405
cg07810884	19	16178099	cg08461451	19	2295092	cg22163535	18	6411387
cg07831553	19	48796974	cg08461576	4	1.02E+08	cg22212560	11	65175482
cg07837852	1	1.99E+08	cg08474901	10	13092035	cg22223402	11	1.14E+08
cg07855465	17	79377665	cg22620071	5	1.13E+08	cg22229114	7	212112
cg07855639	22	37959465	cg22623319	10	1.12E+08	cg22237300	6	52171973
cg07857040	16	1582219	cg22630169	19	13108217	cg22240717	21	44573947
cg07900958	22	49139512	cg22660299	17	46668715	cg22264436	17	41836423
cg07912161	6	36929838	cg22672431	19	1854549	cg22301418	13	1.14E+08
cg07934321	14	40887312	cg22691926	3	27156148	cg22307331	8	61326619
cg07937453	6	89296807	cg22715906	17	4098178	cg22326328	7	1.51E+08
cg07948085	10	9805340	cg22734058	2	2.39E+08	cg22343275	11	2206073
cg07988171	19	16199419	cg22776912	15	81630789	cg22410826	13	1.1E+08
cg07989293	2	1.61E+08	cg22779896	4	6770554	cg22471075	22	49765229
cg08012275	11	1464300	cg22796265	7	24963055	cg22493616	7	1E+08
cg08094206	6	30122523	cg22807822	19	11289365	cg22496559	3	1.96E+08
cg08118908	16	15787920	cg22821300	15	79296585	cg22506548	1	2996949
cg22588983	5	38783142	cg22828884	3	71217277	cg22509679	12	10379548
cg22603628	8	1642625	cg22883290	2	1.28E+08	cg22525581	12	54439112
cg22608619	1	10703231	cg22908922	14	1.06E+08	cg22528123	15	67417964
cg22618405	2	86038802	cg22923006	13	1.14E+08	cg22560020	1	2.47E+08
cg22946774	11	26581996	cg22946147	7	88425148	cg22561592	15	88320395
cg22580512	12	1.25E+08						

Supplementary Table 2: 267 hotspot mutations associated with malignant hematologic disorders

ABCB1	BRIP1	CREBBP	EPOR	HAX1	KLHL6	NFKBIE	PRF1	SF3B1	TMEM30A
ABL1	BTG1	CRLF2	ETNK1	HLA-A	KMT2A	NOTCH1	PRKCB	SGK1	TMSBX
ANKRD26	BTG2	CSF1R	ETV6	HLAC	KMT2B	NOTCH2	PRKD2	SH2B3	TNFAIP3
APC	BTK	CSF3R	EZH2	HLA-DMB	KMT2C	NOTCH3	PRKDC	SH2D1A	TNFRSF14
ARID1A	CALR	CSMD1	FAM46C	HNRNPK	KMT2D	NOTCH4	PRPF8	SMARCA4	TOX
ARID1B	CARD11	CSNK1A1	FAS	HRAS	KRAS	NPM1	PRPS1	SMC1A	TP53
ARID2	CBL	CTCF	FAT1	HUWE1	KRT20	NRAS	PSMB5	SMC3	TPMT
ARID5B	CBLB	CUX1	FAT4	HVCN1	LCOR	NT5C2	PTEN	SMO	TRAF3
ASXL1	CBLC	CXCR4	FBXO11	ID3	LM02	P2RY8	PTPN1	SOCS1	U2AF1
ASXL2	CCND1	CYLD	FGFR3	IDH1	LTB	PALB2	PTPN11	SP140	UBE2A
ATG2B	CCND3	DDX3X	FLT3	IDH2	LYN	PAX5	PTPRD	SPEN	UBR5
ATM	CCR4	DDX41	FOXO1	IGLL5	MAP2K1	PBXW7	RAD21	SRP72	USP7
ATRX	CD28	DIS3	FYN	IKZF1	MAPK1	PDGFRA	RASA2	SRSF2	VAV1
B2M	CD58	DKC1	GAB2	IKZF2	MAX	PDGFRB	RB1	STAG2	VHL
BACH2	CD79A	DNM2	GATA1	IKZF3	MCL1	PDS5B	RBBP6	STAT3	WHSC1
BCL10	CD79B	DNMT3A	GATA2	IL7R	MED12	PHF6	RELN	STAT5B	WT1
BCL2	CDC25C	DNMT3B	GATA3	IRF2BP2	MEF2B	PIGA	RHOA	STAT6	XBP1
BCL6	CDKN1A	DTX1	GFI1	IRF4	MFHAS1	PIK3CA	RPL10	SUFU	XPO1
BCL7A	CDKN1B	DUSP2	GNA13	IRF8	MPL	PIK3CD	RRAGC	SUZ12	ZAP70
BCOR	CDKN2A	EBF1	GNAI2	ITPKB	MTOR	PIK3R1	RUNX1	SYK	ZBTB7A
BCORL1	CDKN2B	EED	GNAS	JAK1	MYC	PIM1	SAMHD1	TAL1	ZFP36L1
BIRC3	CDKN2C	EGFR	GNB1	JAK2	MYCN	PIM2	SBDS	TBL1XR1	ZMYM3
BLM	CEBPA	EGLN1	GSKIP	JAK3	MYD88	PLCG1	SETBP1	TCF3	ZNF292
BPGM	CHD2	EGR1	H1-2	JUNB	MYOM2	PLCG2	SETD1B	TERC	ZRSR2
BRAF	CHD8	ELANE	H1-3	KDM6A	NF1	POT1	SETD2	TERT	
BRCA1	CIITA	EP300	H1-4	KIT	NFE2	PPM1D	SETDB1	TET1	
BRCA2	CNOT3	EPHA7	H1-5	KLF2	NFKBIA	PRDM1	SF1	TET2	

Supplementary Table 3: The 100% PPC of drug sensitivity for inhibition rate test in vitro

Drug	Concentration （nM）
Daunorubicin	995.83
Cytarabine	3468.4
Homoharringtonine	381.22
Cyclophosphamide	196612
Decitabine	3233.9
Etoposide	27695
Amsacrine	10000
ABT-199	600
Sorafenib	10541
Glasdegib	10000
Cladribine	600
Enasidenib	10000
Azacytidine	1660.1
Idarubicin	34.071
Mitoxantrone	3194.7
Aclacinomycin	10000

Supplementary Table 4: Independent test between UAMOCS subtype and 65 mutations

Gene (Mutated)	TMB	UAMOCS1	UAMOCS2	UAMOCS3	P value
DNMT3A	20 (22%)	6 (18.8%)	8 (33.3%)	6 (17.6%)	3.30e-01
IDH1	9 (10%)	4 (12.5%)	2 (8.3%)	3 (8.8%)	8.25e-01
PTPN11	5 (6%)	1 (3.1%)	2 (8.3%)	2 (5.9%)	8.48e-01
NPM1	23 (26%)	1 (3.1%)	11 (45.8%)	11 (32.4%)	2.58e-04
PHF6	4 (4%)	2 (6.2%)	1 (4.2%)	1 (2.9%)	8.31e-01
TTN	7 (8%)	3 (9.4%)	2 (8.3%)	2 (5.9%)	8.90e-01
IDH2	12 (13%)	8 (25.0%)	3 (12.5%)	1 (2.9%)	2.63e-02
RUNX1	12 (13%)	10 (31.2%)	2 (8.3%)	0 (0.0%)	2.22e-04
PLCE1	3 (3%)	2 (6.2%)	1 (4.2%)	0 (0.0%)	3.67e-01
PCLO	3 (3%)	0 (0.0%)	2 (8.3%)	1 (2.9%)	2.65e-01
ASXL1	3 (3%)	3 (9.4%)	0 (0.0%)	0 (0.0%)	5.94e-02
NRAS	6 (7%)	0 (0.0%)	2 (8.3%)	4 (11.8%)	1.61e-01
CEBPA	8 (9%)	2 (6.2%)	1 (4.2%)	5 (14.7%)	4.53e-01
SMC1A	3 (3%)	1 (3.1%)	2 (8.3%)	0 (0.0%)	1.86e-01
FLT3	26 (29%)	3 (9.4%)	7 (29.2%)	16 (47.1%)	2.74e-03
TP53	5 (6%)	5 (15.6%)	0 (0.0%)	0 (0.0%)	5.55e-03
RAD21	4 (4%)	0 (0.0%)	1 (4.2%)	3 (8.8%)	2.45e-01
KIT	5 (6%)	0 (0.0%)	0 (0.0%)	5 (14.7%)	1.19e-02
U2AF1	5 (6%)	4 (12.5%)	1 (4.2%)	0 (0.0%)	4.75e-02
TET2	8 (9%)	3 (9.4%)	3 (12.5%)	2 (5.9%)	7.35e-01
KRAS	5 (6%)	3 (9.4%)	2 (8.3%)	0 (0.0%)	1.80e-01
PARP14	3 (3%)	0 (0.0%)	2 (8.3%)	1 (2.9%)	2.65e-01
FCGBP	4 (4%)	2 (6.2%)	1 (4.2%)	1 (2.9%)	8.31e-01
PKD1L2	3 (3%)	1 (3.1%)	0 (0.0%)	2 (5.9%)	7.78e-01
SMC3	3 (3%)	1 (3.1%)	1 (4.2%)	1 (2.9%)	1.00e+00
WT1	8 (9%)	3 (9.4%)	0 (0.0%)	5 (14.7%)	1.39e-01
DST	3 (3%)	0 (0.0%)	2 (8.3%)	1 (2.9%)	2.65e-01
LRP1B	3 (3%)	1 (3.1%)	1 (4.2%)	1 (2.9%)	1.00e+00
GPR112	5 (6%)	1 (3.1%)	1 (4.2%)	3 (8.8%)	6.29e-01
SPEN	3 (3%)	1 (3.1%)	2 (8.3%)	0 (0.0%)	1.86e-01
MUC16	4 (4%)	1 (3.1%)	2 (8.3%)	1 (2.9%)	6.73e-01
RNF213	3 (3%)	1 (3.1%)	1 (4.2%)	1 (2.9%)	1.00e+00
COL12A1	4 (4%)	1 (3.1%)	0 (0.0%)	3 (8.8%)	4.47e-01
NEB	3 (3%)	0 (0.0%)	1 (4.2%)	2 (5.9%)	4.81e-01
LRFN3	3 (3%)	0 (0.0%)	2 (8.3%)	1 (2.9%)	2.65e-01
BOLA2	3 (3%)	3 (9.4%)	0 (0.0%)	0 (0.0%)	5.94e-02
MAP2	3 (3%)	1 (3.1%)	1 (4.2%)	1 (2.9%)	1.00e+00
CHD4	3 (3%)	1 (3.1%)	1 (4.2%)	1 (2.9%)	1.00e+00
PKHD1	3 (3%)	1 (3.1%)	1 (4.2%)	1 (2.9%)	1.00e+00
BSN	4 (4%)	3 (9.4%)	1 (4.2%)	0 (0.0%)	1.35e-01

Gene (Mutated)	TMB	UAMOCS1	UAMOCS2	UAMOCS3	P value
DNAH11	3 (3%)	0 (0.0%)	0 (0.0%)	3 (8.8%)	1.10e-01
ATF7IP	3 (3%)	0 (0.0%)	2 (8.3%)	1 (2.9%)	2.65e-01
NF1	4 (4%)	2 (6.2%)	1 (4.2%)	1 (2.9%)	8.31e-01
APOB	3 (3%)	1 (3.1%)	0 (0.0%)	2 (5.9%)	7.78e-01
GATA2	4 (4%)	0 (0.0%)	2 (8.3%)	2 (5.9%)	3.06e-01
GABBR1	3 (3%)	1 (3.1%)	2 (8.3%)	0 (0.0%)	1.86e-01
SF3B1	3 (3%)	2 (6.2%)	0 (0.0%)	1 (2.9%)	6.25e-01
SYNE1	3 (3%)	0 (0.0%)	1 (4.2%)	2 (5.9%)	4.81e-01
USP9X	3 (3%)	0 (0.0%)	2 (8.3%)	1 (2.9%)	2.65e-01
MYCBP2	3 (3%)	1 (3.1%)	1 (4.2%)	1 (2.9%)	1.00e+00
DAAM2	3 (3%)	2 (6.2%)	1 (4.2%)	0 (0.0%)	3.67e-01
PLSCR1	3 (3%)	0 (0.0%)	1 (4.2%)	2 (5.9%)	4.81e-01
EP300	3 (3%)	0 (0.0%)	0 (0.0%)	3 (8.8%)	1.10e-01
KIF15	3 (3%)	0 (0.0%)	1 (4.2%)	2 (5.9%)	4.81e-01
ST18	3 (3%)	1 (3.1%)	1 (4.2%)	1 (2.9%)	1.00e+00
LRP2	3 (3%)	1 (3.1%)	1 (4.2%)	1 (2.9%)	1.00e+00
BRWD1	4 (4%)	2 (6.2%)	2 (8.3%)	0 (0.0%)	1.89e-01
SLC16A7	3 (3%)	1 (3.1%)	0 (0.0%)	2 (5.9%)	7.78e-01
ZNF253	3 (3%)	0 (0.0%)	0 (0.0%)	3 (8.8%)	1.10e-01
RALGPS2	3 (3%)	2 (6.2%)	0 (0.0%)	1 (2.9%)	6.25e-01
CHL1	3 (3%)	1 (3.1%)	2 (8.3%)	0 (0.0%)	1.86e-01
GDI2	3 (3%)	1 (3.1%)	2 (8.3%)	0 (0.0%)	1.86e-01
HPS3	4 (4%)	1 (3.1%)	2 (8.3%)	1 (2.9%)	6.73e-01
BPIFC	4 (4%)	1 (3.1%)	0 (0.0%)	3 (8.8%)	4.47e-01
ITGA1	3 (3%)	1 (3.1%)	1 (4.2%)	1 (2.9%)	1.00e+00

Supplementary Table 5: ***Subtype specific genes for*** **UAMOCS**

UAMOCS1-Sepcific	UAMOCS2-Sepcific	UAMOCS3-Sepcific
APP	VCAN	IGLL1
BAALC	HK3	MPO
PROM1	MPEG1	CD96
CD34	LYZ	GTSF1
SDK2	CES1	C1QTNF4
SHANK3	CDA	LPO
NPDC1	FBP1	SUCNR1
F2RL1	CD163	ZBED2
MYCT1	S100A9	POU4F1
H1F0	CD1D	CLEC11A
CHRDL1	CD14	CPA3
NPR3	LILRB4	HPGDS
PEAR1	MAFB	CYTL1
ABO	MS4A6A	DLC1
MN1	KLF4	DPY19L2
ADAMTS10	S100A8	ST18
SETBP1	LINC00482	MACC1
PAWR	HNMT	WT1
SCRN1	CSTA	NTNG2
HEMGN	SIRPB1	LTK
SPON1	NFAM1	S100B
CALN1	TLR8	MDFI
BEND4	IL1RN	MEST
DNTT	S100A12	GGT5
ZNF521	CPM	ACSM1
CD109	RNASE6	AMN
C1orf21	ANXA5	PDLIM1
HBB	SIGLEC9	GATA2
HOPX	RBM47	MYCN
PXDN	SCPEP1	ZNF804A
MLLT3	CYBB	LPAR4
SPRY1	PLBD1	QPRT

DCHS1	SERPINB2	CRNDE
ITGA9	CX3CR1	CPXM1
STARD9	BASP1	CCL1
GBP4	DEFB1	ACSM3
ABLIM1	LGALS2	RTN4R
EHD2	SIGLEC7	DEPDC7
TMIGD2	LDLRAD3	STON2
GPR173	MS4A14	KCNQ5
CALCRL	CD300C	EPX
FAM171B	SCIMP	MGST1
LRP6	TLR4	MICALL2
CDC42BPA	FCER1G	IRX1
IFITM3	FGL2	WT1-AS
PLSCR4	SERPINA1	ITPKA
CD200	CCL23	PTGDR2
CRHBP	SLC22A15	SOX4
SPARC	ARHGEF10L	EVA1B
INPP4B	MS4A7	DEPTOR

Supplementary Table 6: Clinical characteristics in ND ihCAMs-AML cohort under UAMOCS

	level	Overall	UAMOCS1	UAMOCS2	UAMOCS3	Overall P-value	UAMOCS1 vs UAMOCS2 P-value	UAMOCS1 vs UAMOCS3 P-value	UAMOCS1 vs UAMOCS3 P-value
N		80	18	30	32				
Age (%)	<=55	65 (81.2)	11 (61.1)	25 (83.3)	29 (90.6)	0.035	0.168	0.033	0.633
	>55	15 (18.8)	7 (38.9)	5 (16.7)	3 (9.4)				
Sex (%)	female	37 (46.2)	5 (27.8)	16 (53.3)	16 (50.0)	0.196	0.153	0.219	0.993
	male	43 (53.8)	13 (72.2)	14 (46.7)	16 (50.0)				
HSCT (%)	No	59 (73.8)	14 (77.8)	22 (73.3)	23 (71.9)	0.900	1.000	0.904	1.000
	Yes	21 (26.2)	4 (22.2)	8 (26.7)	9 (28.1)				
Status (%)	alive	59 (73.8)	7 (38.9)	21 (70.0)	31 (96.9)	<0.001	0.070	<0.001	0.011
	Dead	21 (26.2)	11 (61.1)	9 (30.0)	1 (3.1)				
FAB Classification (%)	M0	1 (1.2)	1 (5.6)	0 (0.0)	0 (0.0)	<0.001	0.473	0.007	<0.001
	M1	4 (5.0)	1 (5.6)	1 (3.3)	2 (6.2)				
	M2	28 (35.0)	4 (22.2)	3 (10.0)	21 (65.6)				
	M4	16 (20.0)	3 (16.7)	7 (23.3)	6 (18.8)				
	M5	31 (38.8)	9 (50.0)	19 (63.3)	3 (9.4)				
Cytogenetic Group (%)	Adverse	13 (16.2)	6 (33.3)	5 (16.7)	2 (6.2)	0.085	0.225	0.016	0.382
	Favorable	16 (20.0)	1 (5.6)	6 (20.0)	9 (28.1)				
	Normal	51 (63.7)	11 (61.1)	19 (63.3)	21 (65.6)				
Cytogenetic Abnormality (%)	complex	4 (5.0)	2 (11.1)	0 (0.0)	2 (6.2)	0.001	0.157	0.033	0.001
	del(7)	1 (1.2)	1 (5.6)	0 (0.0)	0 (0.0)				
	inv(16)	8 (10.0)	1 (5.6)	6 (20.0)	1 (3.1)				
	MLL rearrangement	8 (10.0)	3 (16.7)	5 (16.7)	0 (0.0)				
	none	51 (63.7)	11 (61.1)	19 (63.3)	21 (65.6)				
	t(8;21)	8 (10.0)	0 (0.0)	0 (0.0)	8 (25.0)				
ELN (%)	adverse	27 (33.8)	13 (72.2)	10 (33.3)	4 (12.5)	<0.001	0.023	<0.001	0.037
	favorable	39 (48.8)	2 (11.1)	13 (43.3)	24 (75.0)				
	intermediate	14 (17.5)	3 (16.7)	7 (23.3)	4 (12.5)				
Response (%)	CR	41 (51.2)	5 (27.8)	18 (60.0)	18 (56.2)	0.074	0.062	0.100	0.967
	PR+NR	39 (48.8)	13 (72.2)	12 (40.0)	14 (43.8)				

Note: The response refers to the assessment of response following the completion of induction chemotherapy. CR: complete remission; NR: no remission; PR: partial remission.

Supplementary Table 7: Clinical data regrading to CBF-AML in ihCAMs-AML cohort

**Clinical Features**	**Class**	**UAMOCS2**<br>（N=9）	**UAMOCS3**<br>(N=10)	***P*-value**
Age (%)	<=55	8 (88.9)	10 (100.0)	0.474
	>55	1 (11.1)	0 (0.0)	
Sex (%)	female	3 (33.3)	4 (40.0)	1
	male	6 (66.7)	6 (60.0)	
FAB Classification (%)	M2	2 (22.2)	9 (90.0)	0.005
	M4	7 (77.8)	1 (10.0)	
Fusion Gene Abnormality (%)	CBFβ::MYH11	7 (77.8)	1 (10.0)	0.005
	RUNX1::RUNX1T1	2 (22.2)	9 (90.0)	
HSCT (%)	No	8 (88.9)	9 (90.0)	1
	Yes	1 (11.1)	1 (10.0)	
AML-MR (%)	Mutant	0 (0.0)	5 (50.0)	0.033
	Wild	9 (100.0)	5 (50.0)	
Relapse rate (%)	No	7 (88.9)	4 (30.0)	0.04
	Yes	2 (22.2)	6 (60.0)	

Supplementary Table 8: The 100% PPC inhibition rate of three clusters under UAMOCS

Drug	UAMOCS1	UAMOCS2	UAMOCS3	p
Aclacinomycin	43.3959571	46.4630696	48.095776	0.9315
Daunorubicin	41.7206357	56.617813	66.288004	0.1248
Idarubicin	39.2529714	45.173687	48.828528	0.6201
Mitoxantrone	45.3685214	56.9396739	64.695424	0.0873
Cytarabine	45.9265	60.3423	67.264788	0.1953
Fludarabine	45.2735929	55.0821217	57.928252	0.4047
Cladribine	44.80445	59.3690087	60.648128	0.2184
Enasidenib	20.1259714	18.7800652	19.086808	0.9421
Homoharringtonine	39.7033143	59.3339609	68.56184	0.1434
Cyclophosphamide	23.8384857	24.6206522	33.948196	0.3925
Decitabine	17.1067357	15.827787	22.117396	0.7667
Azacytidine	39.8136	46.727887	61.928604	0.0468
Etoposide	23.1227571	29.7772261	35.679784	0.3203
Amsacrine	26.4812143	35.8113913	55.148968	0.0025
ABT-199	25.8702286	30.3818783	46.058316	0.0055
Glasdegib	18.4096429	22.438913	19.208656	0.7459
Sorafenib	24.8236786	23.6063478	29.167756	0.5041

Supplementary Table 9: The core genes in previous established prognostic models and our model

Gene score	gene panel	Reference
LSC 17 score	DNMT3B, GPR56, CD34, SOCS2, SMIM24, FAM30A, AKR1C3, EMP1, CDK6, CPXM1, ZBTB46, NYNRIN, LAPTM4B, MMRN1, KIAA0125, DPYSL3, ARHGAP22	(1)
5 gene score	CALCRL, DOCK1, LRCH4, FCHO2, PLA2G4A	(2)
24 gene score	ALS2CR8, ANGEL1, ARL6IP5, BSPRY, BTBD3, C1RL, CPT1A, DAPK1, ETFB, FGFR1, HEATR6, LAPTM4B, MAP7, NDFIP1, PBX3, PLA2G4A, PLOD3, PTP4A3, SLC25A12, SLC2A5, TMEM159, TRIM44, TRPS1, VAV3	(3)
UAMOCS	UAMOCS1:<br>F2RL1, MN1, GBP4, ABLIM1, PLSCR4<br>UAMOCS2:<br>VCAN, HK3, MPEG1, CD1D, CD14, LGALS2<br>UAMOCS3:<br>IGLL1, CD96, SUCNR1, PDLIM1, ACSM3	This research

Supplementary Figure

Supplementary Fig. 1

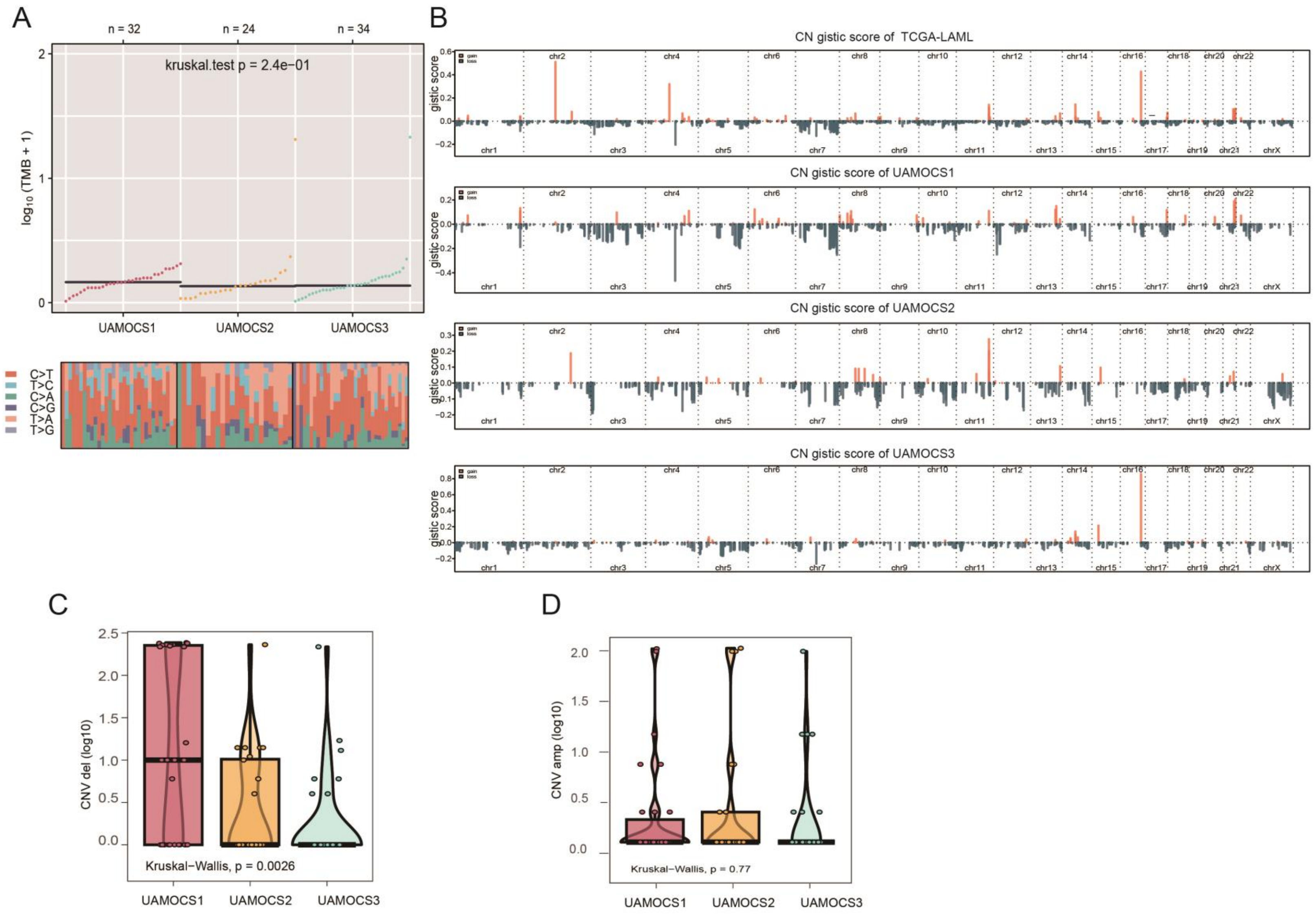

Supplementary Fig. 2

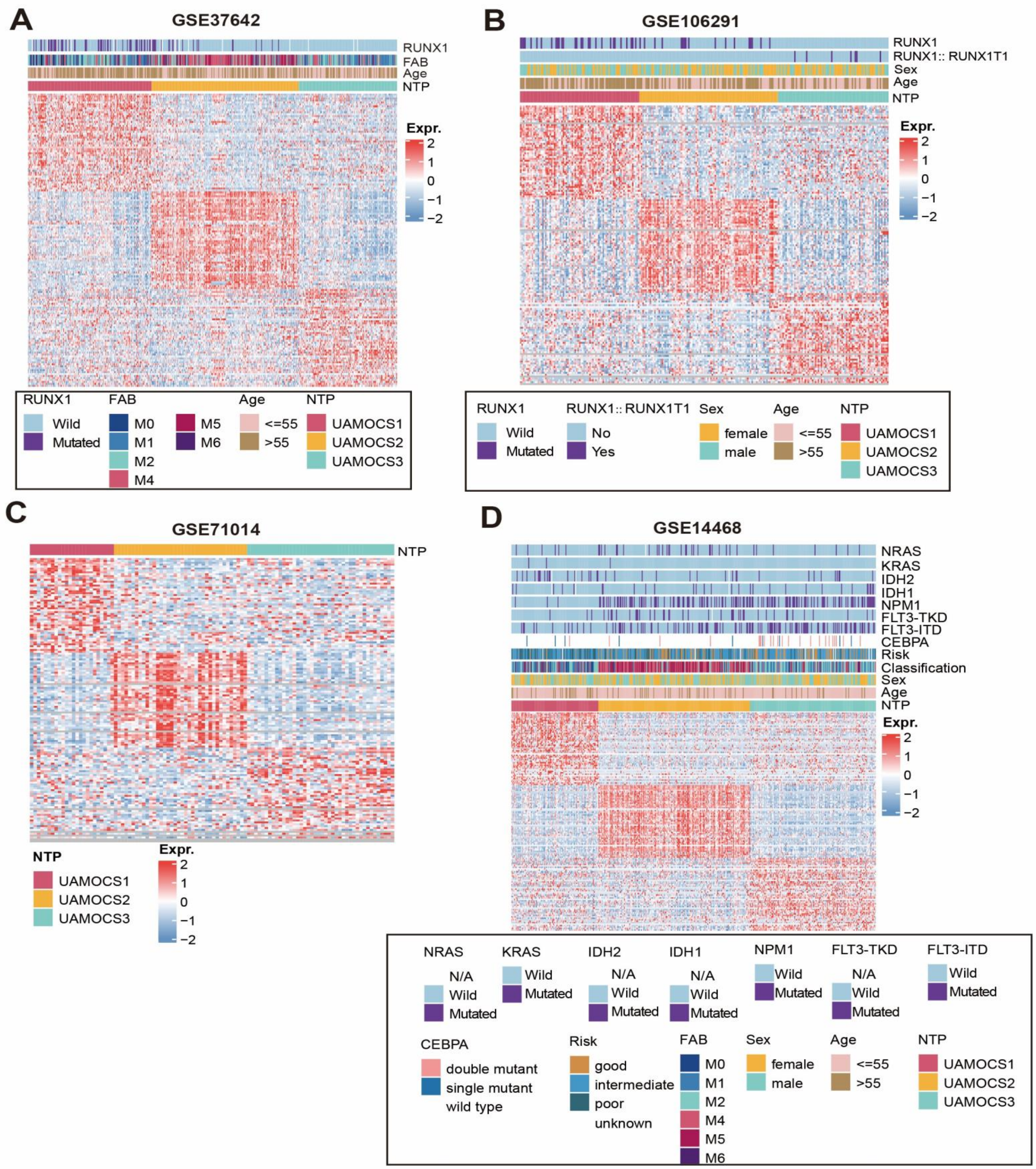

Supplementary Fig. 3

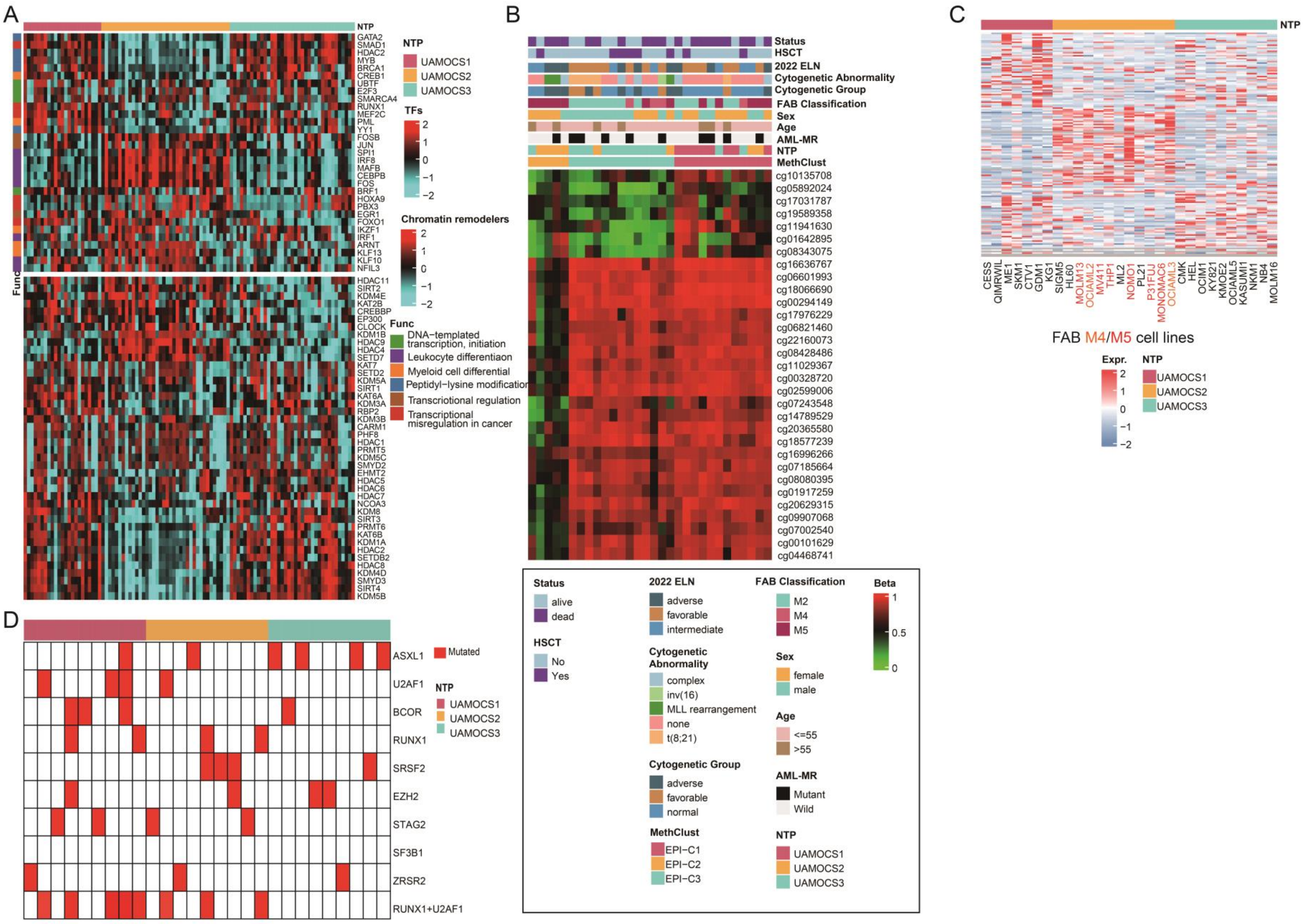

Supplementary Fig. 4

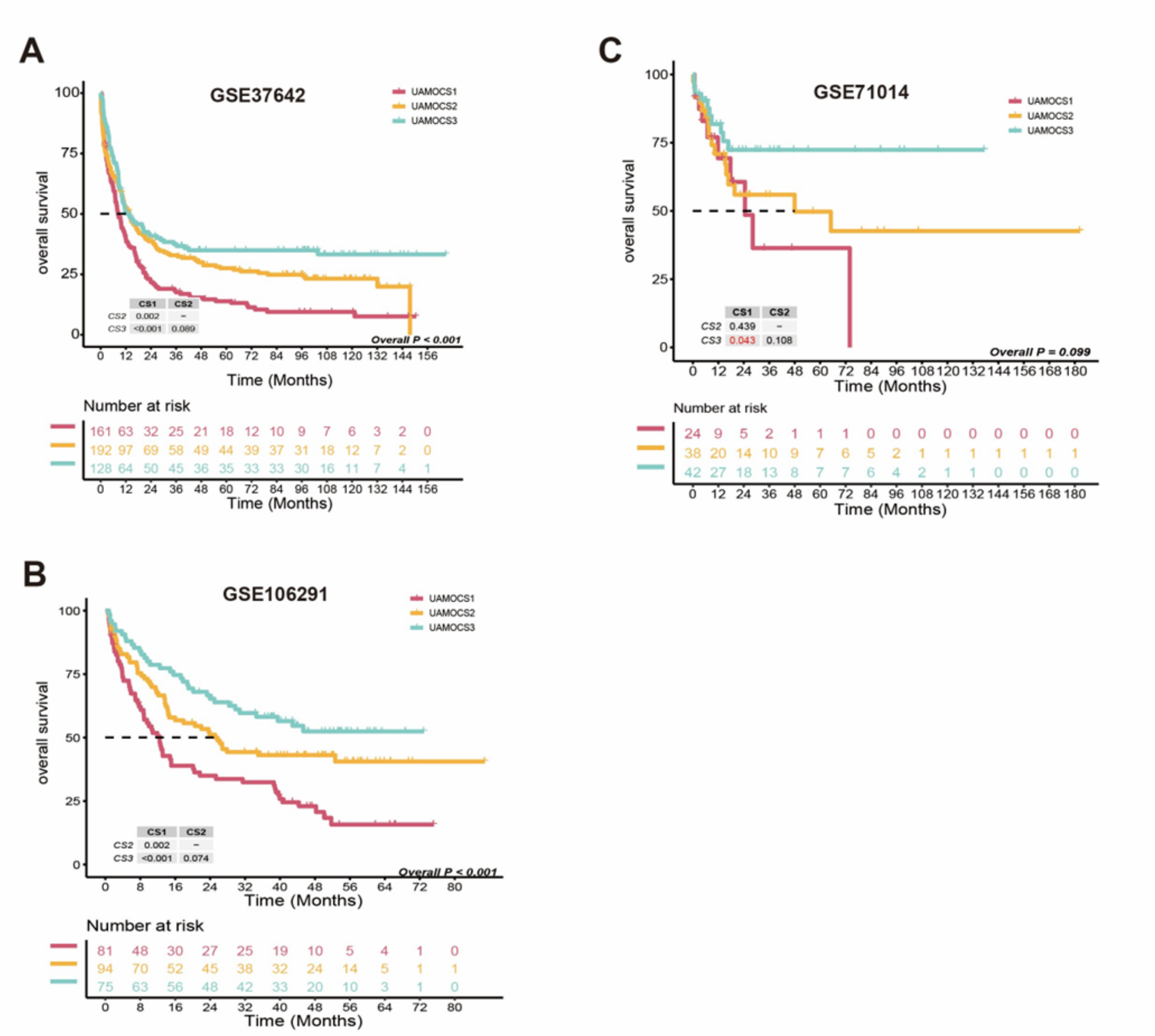

Supplementary Fig. 5

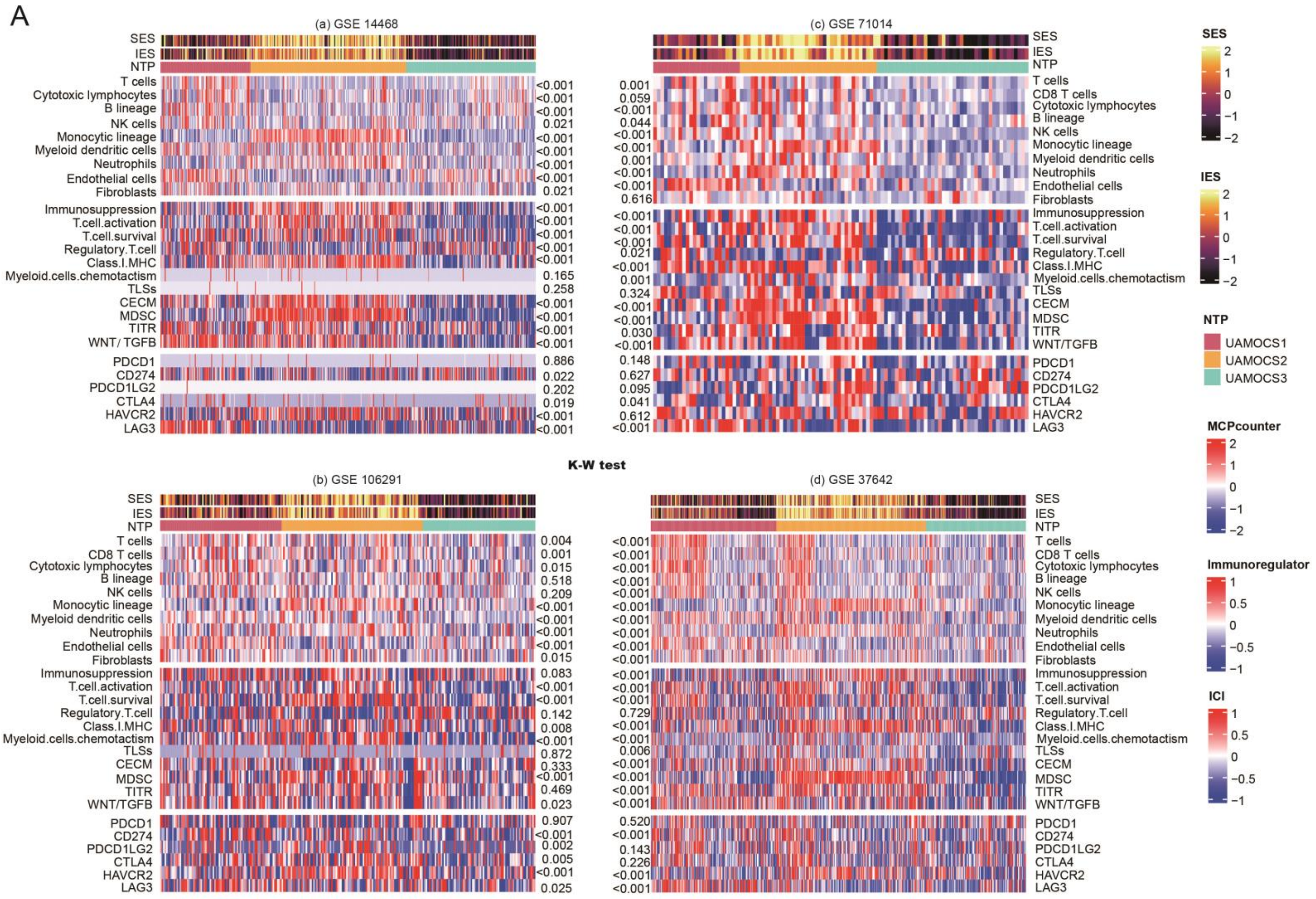

Supplementary Fig. 6

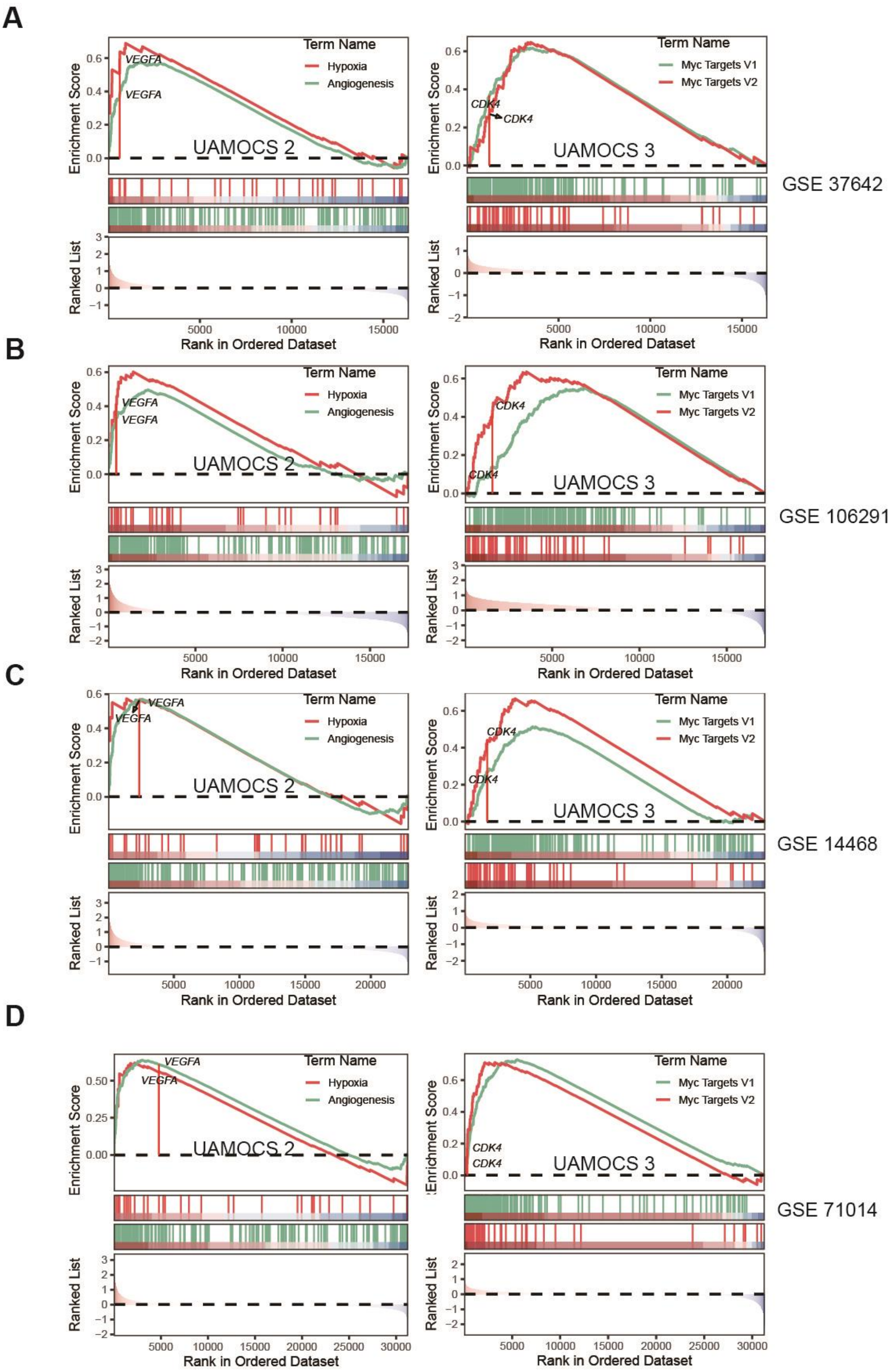

Supplementary Figures Legend

Supplementary Fig. 1 | Detectable molecular and cytogenetic abnormality of three UAMOCS subtypes in TCGA-LAML cohort

A The distributions of TMB burden with detailed alteration bases among three UAMOCS subtypes.

B The copy number alterations (amplifications and deletions) distribution among the 22 chromosomes in the three subgroups under UAMOCS in TCGA. CN： copy number

C The violin graph depicting the proportion of Genome deletions across three subtypes.

D The violin graph depicting the proportion of Genome amplification across three subtypes.

Supplementary Fig. 2 | Characteristics of four public datasets as externally validated cohort through NTP algorithm

A-D. Heatmaps illustrating UAMOCS across four independent GEO Datasets. Specifically, 150 subtype-specific gene expression for shaping UAMOCS1-3 were utilized to develop the UAMOCS classifier for each dataset, including GSE 37642 **(A**), GSE 106291 (**B**), GSE 14468 (**C**), and GSE 71014 (**D**) through NTP algorithm.

Supplementary Fig. 3 | Validation of UAMOCS within the ihCAMs-AML Cohort

A. Heatmap showing distinct regulon activity patterns across the three UAMOCS subtypes, with 31 transcription factors (TFs) in the top panel and chromatin remodeling regulators in the bottom panel, derived from comparisons in the ihCAMs-AML cohort.

B. The consensus heatmap highlighting methylation profiles within the ihCAMs-AML cohort identified by UAMOCS, applying demethylation positions derived from TCGA. Annotations at the right indicate UAMOCS subtypes and relevant clinical factors. HSCT: Hematopoietic Stem Cell Transplantation; ELN: European LeukemiaNet; AML-MR: Acute Myeloid Leukemia with Myelodysplasia-Related Changes; Beta: methylation beta values

C. Heatmap showing UAMOCS subtyping in the Cancer Cell Line Encyclopedia (CCLE) database,

validated using the NTP algorithm. The heatmap displays the expression of 150 subtype-specific genes defining UAMOCS1-3, which were used to develop the UAMOCS classifier and reveal distinct subtype clustering within the CCLE dataset.

D. Heatmap presenting the landscape of eight AML-MR mutations across three UAMOCS subtypes.

Supplementary Fig. 4 | The prognostic value of UAMOCS across different datasets

A-C The Kaplan–Meier curve compares overall survival (OS) across the three molecular subtypes in the GSE37642 (**A**), GSE 106291 (**B**) and GSE 71014 (**C**). Overall P: Fisher Exact Test; pairwise P: Log-Rank test.

Supplementary Fig. 5 | Recognition of UAMOCS immune phenotype across four databases.

A The heatmap showcases the immune landscape of UAMOCS across four databases, serving as a robust validation for the immunological characteristics inherent to UAMOCS three subtypes. GSE 14468(**A**); GSE 106291(**B**); GSE 71014(**C**); GSE 37642(**D**). TITR: tumor−infiltrating Tregs; TLS: tertiary lymphoid structures, C−ECM: cancer−associated extracellular matrix; MDSC: myeloid derived suppressor cells; ICI: and Immune Checkpoint Inhibitors; SES: Stromal Enrichment Score; IES: Immune Enrichment Score

Supplementary Fig. 6 | Distinct upregulated signaling pathways characteristic of UAMOCS subtypes across four databases

A-D: The GSEA plot distinctly highlights hallmark pathways, revealing upregulated pathways unique to UAMOCS2 and UAMOCS3 across four databases, as demonstrated in the left and right panels, respectively. GSE 14468(**A**); GSE 37642(**B**); GSE 106291(**C**); GSE 71014 (**D**).

Supplementary Reference

1. Ng SW, Mitchell A, Kennedy JA, Chen WC, McLeod J, Ibrahimova N, et al. A 17-gene stemness score for rapid determination of risk in acute leukaemia. Nature. 2016;540(7633):433-7.
2. Sha K, Lu Y, Zhang P, Pei R, Shi X, Fan Z, et al. Identifying a novel 5-gene signature predicting clinical outcomes in acute myeloid leukemia. Clin Transl Oncol. 2021;23(3):648-56.
3. Li Z, Herold T, He C, Valk PJ, Chen P, Jurinovic V, et al. Identification of a 24-gene prognostic signature that improves the European LeukemiaNet risk classification of acute myeloid leukemia: an international collaborative study. J Clin Oncol. 2013;31(9):1172-81.
